# Sperm associated antigen 9 promotes oncogenic KSHV-encoded interferon regulatory factor-induced cellular transformation and angiogenesis by activating the JNK/VEGFA pathway

**DOI:** 10.1371/journal.ppat.1008730

**Published:** 2020-08-10

**Authors:** Wan Li, Fei Wang, Jiale Shi, Qi Feng, Yuheng Chen, Xiaoyu Qi, Cong Wang, Hongmei Lu, Zhongmou Lu, Xuemei Jia, Qin Yan, Shou-Jiang Gao, Chun Lu

**Affiliations:** 1 Department of Microbiology, Nanjing Medical University, Nanjing, People’s Republic of China; 2 Department of Gynecology, Women’s Hospital of Nanjing Medical University, Nanjing Maternity and Child Health Hospital, Nanjing Medical University, Nanjing, People’s Republic of China; 3 Key Laboratory of Pathogen Biology of Jiangsu Province, Nanjing Medical University, Nanjing, People’s Republic of China; 4 Department of Pathology, the First Affiliated Hospital of Nanjing Medical University, Nanjing, People’s Republic of China; 5 Department of Obstetrics, the First Affiliated Hospital of Nanjing Medical University, Nanjing, People’s Republic of China; 6 UPMC Hillman Cancer Center, Department of Microbiology and Molecular Genetics, University of Pittsburgh, Pittsburgh, Pennsylvania, United States of America; Heinrich Pette Institute, Leibniz Institute for Experimental Virology, GERMANY

## Abstract

Kaposi’s sarcoma (KS), caused by Kaposi’s sarcoma-associated herpesvirus (KSHV), is a highly angioproliferative disseminated tumor of endothelial cells commonly found in AIDS patients. We have recently shown that KSHV-encoded viral interferon regulatory factor 1 (vIRF1) mediates KSHV-induced cell motility (PLoS Pathog. 2019 Jan 30;15(1):e1007578). However, the role of vIRF1 in KSHV-induced cellular transformation and angiogenesis remains unknown. Here, we show that vIRF1 promotes angiogenesis by upregulating sperm associated antigen 9 (SPAG9) using two *in vivo* angiogenesis models including the chick chorioallantoic membrane assay (CAM) and the matrigel plug angiogenesis assay in mice. Mechanistically, vIRF1 interacts with transcription factor Lef1 to promote SPAG9 transcription. vIRF1-induced SPAG9 promotes the interaction of mitogen-activated protein kinase kinase 4 (MKK4) with JNK1/2 to increase their phosphorylation, resulting in enhanced VEGFA expression, angiogenesis, cell proliferation and migration. Finally, genetic deletion of *ORF*-*K9* from KSHV genome abolishes KSHV-induced cellular transformation and impairs angiogenesis. Our results reveal that vIRF1 transcriptionally activates *SPAG9* expression to promote angiogenesis and tumorigenesis via activating JNK/VEGFA signaling. These novel findings define the mechanism of KSHV induction of the SPAG9/JNK/VEGFA pathway and establish the scientific basis for targeting this pathway for treating KSHV-associated cancers.

## Author summary

Kaposi’s sarcoma-associated herpesvirus (KSHV)-encoded viral interferon regulatory factor 1 (vIRF1) is essential for KSHV-induced angiogenesis and tumorigenesis, but the underlying mechanism has not been elucidated. Sperm associated antigen 9 (SPAG9) is a well-characterized oncoprotein, highly expressed in a variety of human cancer. However, whether SPAG9 could act as a promoter of KS development has not been investigated. Here, we reveal that vIRF1 enhances SPAG9 transcription by forming a transcription factor complex with Lef1. vIRF1-induced SPAG9 upregulation promotes the interaction of mitogen-activated protein kinase kinase 4 (MKK4) with JNK together leading to enhanced JNK phosphorylation. Activation of the JNK pathway induces VEGFA expression, contributing to KSHV-induced cellular transformation and angiogenesis. In summary, this work discovers a novel mechanism of vIRF1 induction of angiogenesis and tumorigenesis, and reveals SPAG9/JNK/VEGFA pathway as a potential target for KS therapy.

## Introduction

Kaposi’s sarcoma (KS), a highly angiogenic and disseminated tumor of endothelial cells commonly found in AIDS patients, is caused by infection of a human oncogenic virus Kaposi’s sarcoma-associated herpesvirus (KSHV). KSHV is a DNA tumor virus of the gammaherpesvirus subfamily, which was identified in an AIDS-associated Kaposi's sarcoma (AIDS-KS) lesion in 1994 [1]. KSHV is also associated with primary effusion lymphoma (PEL), a subset of multicentric Castleman’s disease (MCD), and KSHV-associated inflammatory cytokine syndrome (KICS) [1, 2].

KSHV encodes over 90 open reading frames (ORFs) and at least 25 microRNAs [3]. Similar to other herpesviruses, KSHV life cycle has two phases, latency and lytic replication. The latent phase is characterized by the expression of very limited viral genes. However, once KSHV latently infected cells are reactivated into the lytic phase, all viral genes are expressed, leading to the production of new virions [4]. KSHV encodes several cellular homologues, including viral interferon regulatory factors (vIRFs) [5], viral G protein-coupled receptor (vGPCR) [6], viral cyclin (vCyclin) [7], viral interleukin-6 (vIL-6) [8], viral Bcl-2 (vBcl-2) [9], and viral FLICE inhibitory protein (vFLIP) [10]. These proteins promote cell proliferation and survival, evade immune responses and induce inflammation, contributing to the development and progression of KSHV-induced tumors [11].

KSHV vIRFs (vIRF1 to vIRF4) are encoded by a cluster of ORFs (ORFs-K9, -K11/11.1, -K10.5/10.6, and -K10), which are resided between ORF57 and ORF58 of the viral genome and transcribed in the opposite orientation [12]. vIRFs are homologous to cellular IRFs; however, they do not contain the five tryptophan residues required for DNA-binding [13]. Among them, the vIRF1 protein is comprised of 449 amino acids. Out of all four KSHV vIRFs, vIRF1 has the highest degree of homology with cellular IRFs. It has a C-terminal IRF interaction domain (IAD) and an N-terminal DNA-binding domain (DBD). The sequence of vIRF1 DBD shows a high homology to the DBDs of the cellular IRF3 (41.5%) and IRF7 (38.3%) albeit it only has two of the five conserved tryptophan residues for DNA binding. It is generally considered that vIRF1 does not possess DNA-binding ability [14–16] and it exerts its functions through protein-protein interactions. Nevertheless, in 2007, by using gel shift and chromatin immunoprecipitation assays, Park and colleagues identified the vIRF1-binding consensus sequence located in the promoter region of KSHV K3 (viral E3 ubiquitin ligase), viral dihydrofolate reductase (vDHFR; ORF2) and vIL-6 [17]. In agreement with this finding, vIRF1 overexpression led to the activation of reporters of K3, vDHFR and vIL6 gene promoters, and knockdown of vIRF1 expression in KSHV-positive BCBL-1 cells suppressed vIL-6 transcription [17, 18], suggesting that vIRF1 might play a role in regulating the transcription of certain KSHV genes. More importantly, the crystal structure of vIRF1 DBD in complex with DNA was subsequently revealed [19], providing a strong evidence to support that vIRF1 is a DNA-binding protein, which might act directly on different promoter sequences, as proposed for IRF3.

Sperm associated antigen 9 (SPAG9) encoded by *SPAG9* gene has four isoforms, the longest one is 1,321 amino acids. SPAG9, also called c-Jun-terminal kinase (JNK)-associated leucine zipper protein (JLP) or c-Jun-amino-terminal kinase-interacting protein 4 (JIP4), is a member of the cancer/testis (CT) antigen family. SPAG9 could function as a scaffold protein mediating the interactions of mitogen-activated protein kinase kinases (MKKs) and their targets to activate specific signaling pathways [20, 21]. SPAG9 was highly expressed in a variety of human cancer, including colorectal carcinoma, renal cell carcinoma, cervical, thyroid, and breast cancer [22–26]. SPAG9 expression was also bound up with circulating anti-SPAG9 antibodies, indicating that SPAG9 could be a promising noninvasive serum biomarker for early diagnosis and cancer management [22, 23, 27].

In this work, we demonstrated that vIRF1-Lef1 transcription factor complex was able to bind to SPAG9 promoter to enhance its transcription and expression. High level of SPAG9 was responsible for vIRF1-induced cell proliferation, migration, angiogenesis, and cellular transformation. vIRF1-induced SPAG9 promotes the interaction of mitogen-activated protein kinase kinase 4 (MKK4) with JNK, resulting in an increase of JNK phosphorylation and activation of VEGFA signaling. Moreover, treatment with the JNK inhibitor, SP600125, abolished vIRF1-induced cell oncogenic behaviors. Our novel findings suggest that the SPAG9/JNK/VEGFA pathway induced by vIRF1 is a potential therapeutic target for KSHV-associated malignancies.

## Results

### vIRF1 transcriptionally activates SPAG9

**Fig 1:**
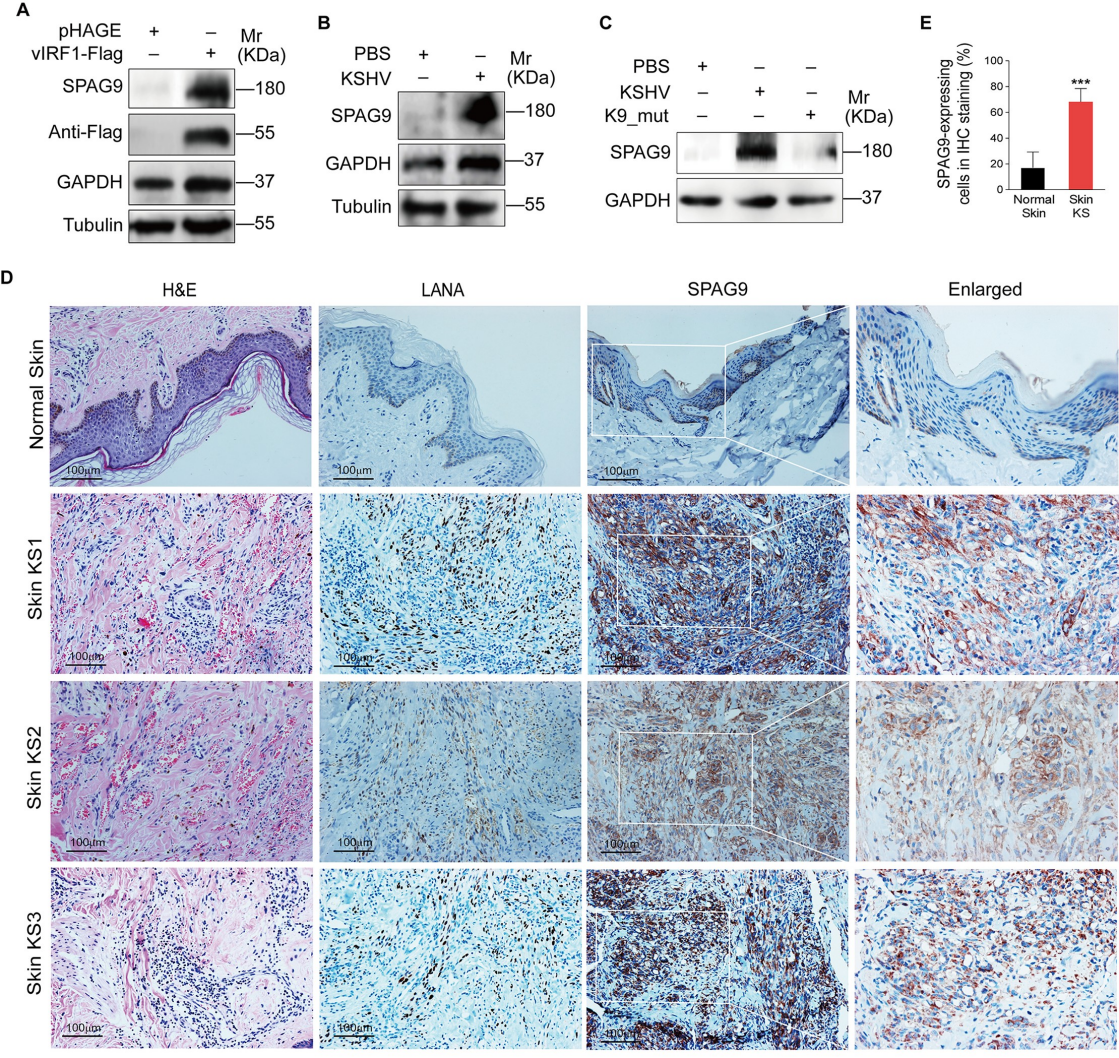
SPAG9 is upregulated in vIRF1-transduced HUVECs and KSHV-infected HUVECs. **(A).** Western-blotting analysis of SPAG9 in HUVECs transduced with lentiviral-vIRF1 or its control lentiviral-pHAGE (MOI of 2). **(B).** Western-blotting analysis of SPAG9 in HUVECs treated with PBS (**PBS**) or infected by KSHV wild-type virus (**KSHV**) (MOI of 3). **(C).** Western-blotting analysis of SPAG9 expression in HUVECs treated with PBS (**PBS**) or infected with wild-type KSHV (**KSHV_WT**) (MOI of 3) or vIRF1 mutant virus (**K9_mut**) (MOI of 3) for 30 h. **(D).** Hematoxylin and eosin (H&E) staining and immunohistochemical staining (IHC) of KSHV LANA, SPAG9 in normal skin, skin KS of patient #1 (Skin KS1), patient #2 (Skin KS2), and patient #3 (Skin KS3). Magnification, ×200, ×400. **(E).** Results were quantified in (**D**). Data were shown as mean ± SD. *** *P* < 0.001, Student's t-test.

SPAG9 is a well-characterized oncoprotein [28], but its role in KS pathogenesis is still unknown. We transduced primary human umbilical vein endothelial cells (HUVECs) with 2 MOI of lentiviral vIRF1. Our previous study showed that at this MOI, vIRF1-transduced HUVECs showed a vIRF1 mRNA level similar to that of KSHV-infected HUVECs with approximately 90% of positive cells at day 2 post-transduction [29]. Western-blotting analysis showed that SPAG9 was strikingly elevated in vIRF1-transduced HUVECs (Fig 1A). Moreover, we infected HUVECs with 3 MOI of wild type KSHV and found that KSHV infection also increased SPAG9 expression (Fig 1B). Deletion of vIRF1 from KSHV genome showed a decrease of SPAG9 protein expression in HUVECs (Fig 1C). Importantly, stronger and more SPAG9-postive cells were observed in KS lesions compared to normal skin tissues (Fig 1D, Fig 1E and S1 Fig).

**Fig 2:**
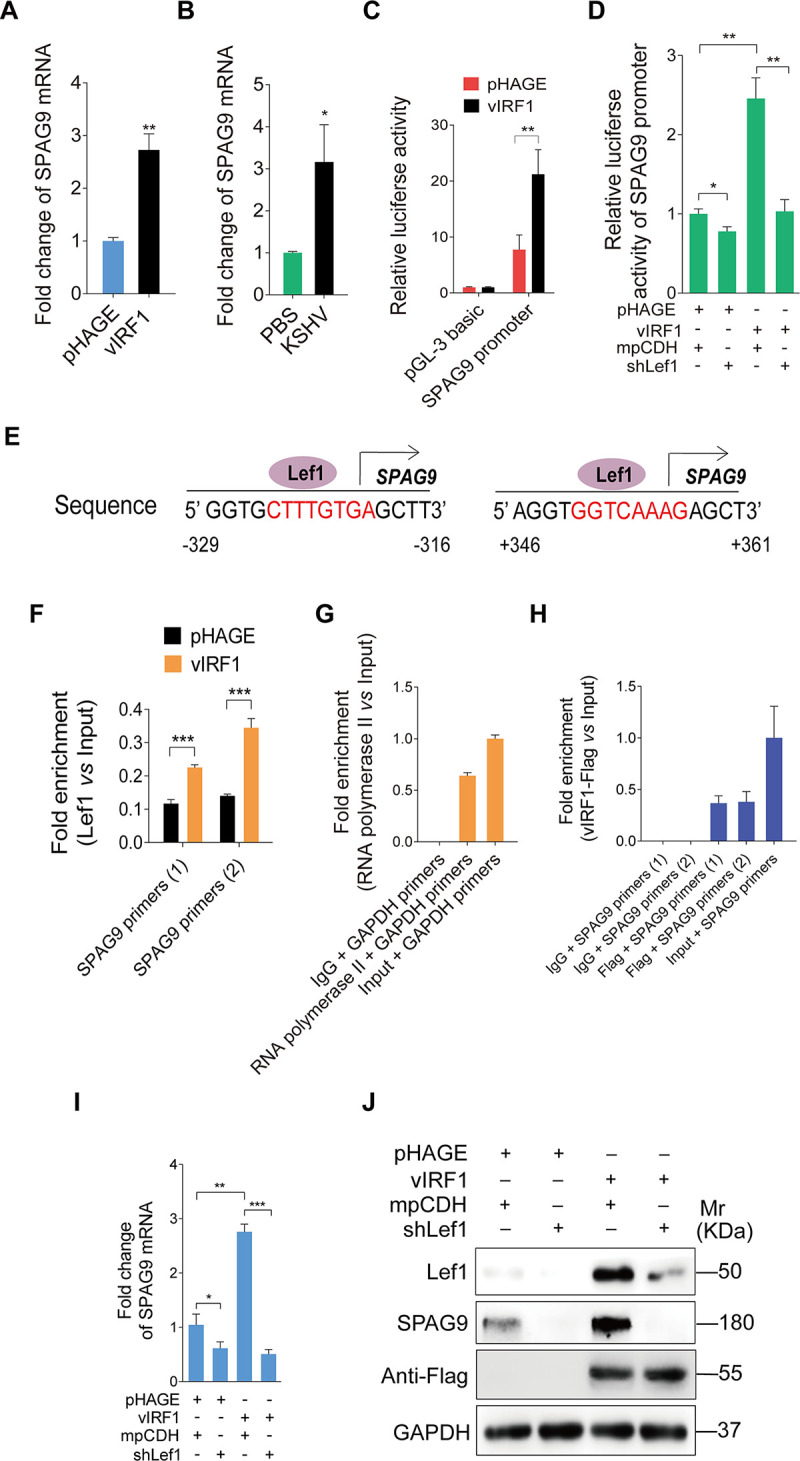
vIRF1 transcriptionally activates SPAG9 by interacting with Lef1. **(A).** RT-qPCR analysis of mRNA level of SPAG9 in HUVECs transduced with lentiviral-vIRF1 or its control lentiviral-pHAGE. **(B).** RT-qPCR analysis of mRNA level of SPAG9 in treated with PBS (**PBS**) or infected by KSHV wild-type virus (**KSHV**) (MOI of 3). **(C).** Luciferase reporter assay of the activity of SPAG9 promoter in HUVECs transduced with lentiviral-vIRF1 or its control lentiviral-pHAGE. **(D).** Luciferase reporter assay of the activity of SPAG9 promoter in vIRF1-expressing HUVECs transduced with a mixture of lentivirus-mediated shRNAs targeting Lef1 (**shLef1**). **(E).** Putative binding sites of Lef1 in the promoter region of SPAG9 gene. **(F).** ChIP assays of SPAG9 promoter. Immunoprecipitation was performed in vIRF1- or pHAGE-transduced HUVECs with anti-Lef1 antibody. Both SPAG9 primers (1) and (2) were used to amplify the sequences of the above two putative binding sites of Lef1 in the region of SPAG9 promoter as described in (**E**). **(G)** and **(H).** ChIP assays of SPAG9 promoter. Immunoprecipitation was performed in vIRF1-transduced HUVECs with anti-Flag antibody. Isotype IgG, anti-RNA polymerase II antibody and amplification of GAPDH promoter were used to examined the work of system. **(I).** RT-qPCR analysis of mRNA expression level of SPAG9 in vIRF1-expressing HUVECs transduced with a mixture of lentivirus-mediated shRNAs targeting Lef1 (**shLef1**). **(J).** Western-blotting analysis of SPAG9 expression in vIRF1-expressing HUVECs transduced with a mixture of lentivirus-mediated shRNAs targeting Lef1 (**shLef1**). Data were shown as mean ± SD. * *P* < 0.05, ** *P* < 0.01, and *** *P* < 0.001, Student's t-test.

We further explored the mechanism by which vIRF1 upregulated SPAG9. A significant increase of SPAG9 mRNA expression was seen in both vIRF1-transduced HUVECs and KSHV-infected cells compared to pHAGE and PBS control cells, respectively (Fig 2A and 2B). There was close relation between vIRF1 and SPAG9 mRNA expression levels during KSHV infection (S2A Fig and S2B Fig). Deletion of vIRF1 diminished SPAG9 mRNA expression (S2C Fig). In a luciferase reporter assay, vIRF1 increased the transcription activity of SPAG9 promoter in a dose-dependent manner (Fig 2C and S3 Fig). In addition, the enhanced transcription activity of SPAG9 promoter was observed in KSHV-infected cells, and deletion of vIRF1 impaired KSHV-induced transcription activity of SPAG9 promoter (S4 Fig). Our recent study reported that vIRF1 interacted with transcription factor Lef1 to bind to the promoter of CDCP1 gene and enhance its transcription and expression [30]. Here, after knocking down the Lef1 with a mixture of lentivirus-mediated short hairpin RNAs (shRNAs), we found that the transcription activity of SPAG9 promoter induced by vIRF1 was dramatically impaired in a luciferase reporter assay (Fig 2D). These data suggested a potential role of vIRF1-Lef1 complex in regulating SPAG9 transcription. Further, two potential binding sites of Lef1 in the SPAG9 promoter were predicted by using the ALGGEN PROMO software (http://alggen.lsi.upc.es/cgi-bin/promo_v3/promo/promoinit.cgi?dirDB=TF_8.3) (Fig 2E). To substantiate the physical interaction of Lef1 with the sequence of SPAG9 promoter, the chromatin immunoprecipitation (ChIP) assay was performed. We found that Lef1 indeed bound to the two predicted sites within the SPAG9 promoter (Fig 2F). Notably, in the presence of vIRF1, Lef1 had increased binding to the SPAG9 promoter (Fig 2F). Using an anti-Flag antibody, we found significant binding of vIRF1 to the SPAG9 promoter (Fig 2G and 2H). As expected, knockdown of Lef1 decreased both mRNA and protein levels of SPAG9 induced by vIRF1 (Fig 2I and 2J). The reduction of both mRNA and protein levels of SPAG9 was also observed in KSHV-infected cells with knockdown of Lef1 (S5A Fig and S5B Fig). These results collectively indicated that vIRF1-Lef1 complex bound to the promoter region of SPAG9 to increase SPAG9 expression.

### vIRF1 upregulates SPAG9 expression to promote angiogenesis, cell proliferation and migration

**Fig 3:**
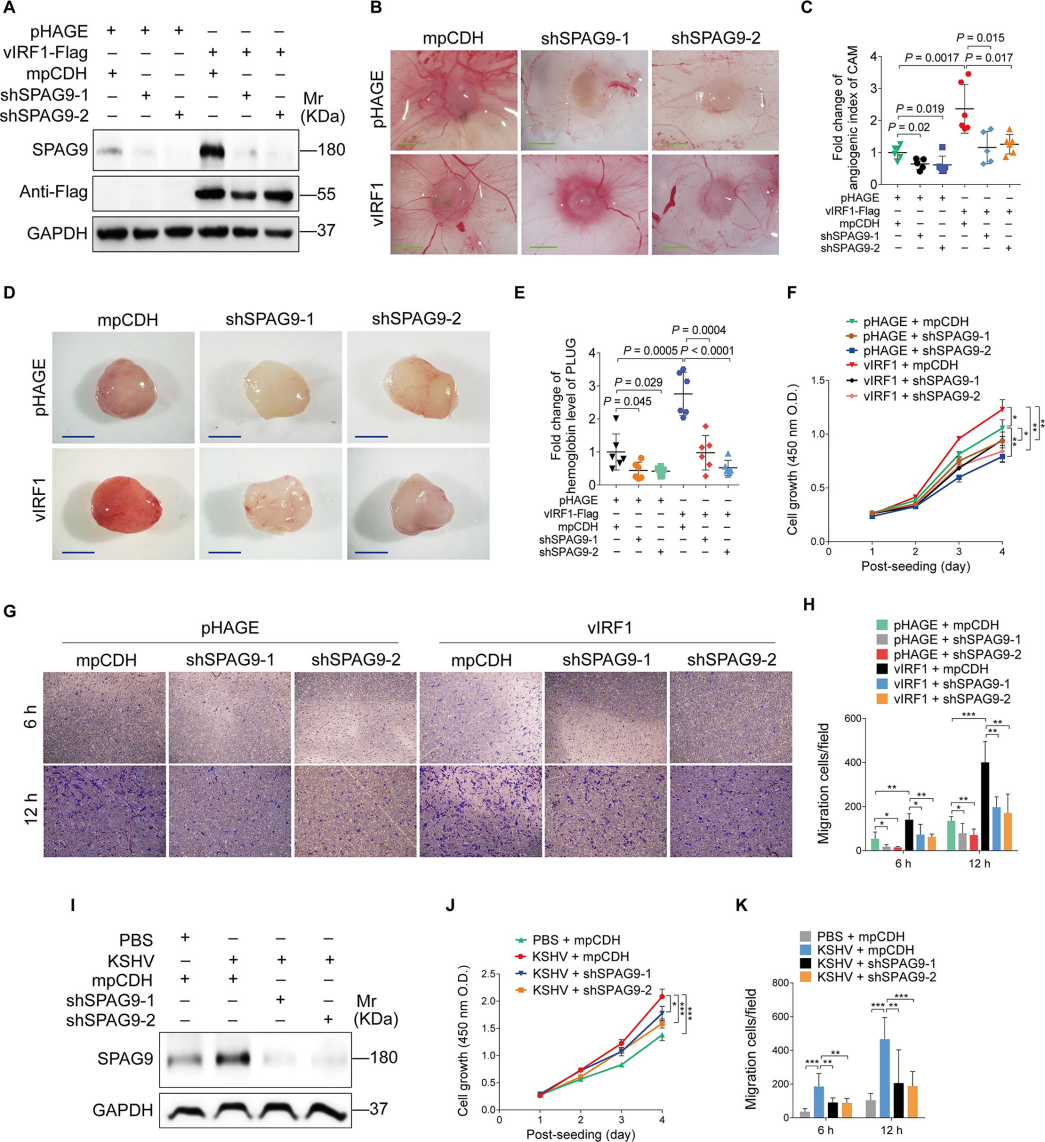
Knockdown of SPAG9 inhibits vIRF1-induced angiogenesis, cell proliferation and migration. **(A).** Western-blotting analysis of SPAG9 expression in vIRF1-expressing HUVECs transduced with lentivirus-mediated No.1 (**shSPAG9-1**) and No. 2 (**shSPAG9-2**) shRNAs targeting SPAG9. **(B).** Lentiviral vIRF1- or its control pHAGE-infected endothelial cell line were transduced with lentivirus-mediated No.1 (**shSPAG9-1**) and No. 2 (**shSPAG9-2**) shRNAs targeting SPAG9, and then were subjected to chicken chorioallantoic membranes (CAMs) assay. Representative images are shown. Magnification, ×100. Scar bars, 40 μm. **(C).** Quantification of CAMs assay described in (**B**). **(D).** Cells treated as in (**B**) were mixed with the high concentration Matrigel, and then were injected into the right flanks of nude mice. Plugs were harvested 10 days after the injection and photographed using stereomicroscope. Representative images of Matrigel plug assay in mice are displayed. **(E).** Quantification of Matrigel plug assay in mice described in (**D**). **(F).** CCK-8 assay of HUVECs treated as in (**A**). **(G).** Transwell migration analysis of HUVECs treated as in (**A**). The migrated and invaded HUVECs were counted at 6 h and 12 h post seeding. **(H).** Quantification of Transwell migration assay described in (**G**). **(I).** Western-blotting analysis of SPAG9 expression in KSHV-infected HUVECs transduced with lentivirus-mediated No.1 (**shSPAG9-1**) and No. 2 (**shSPAG9-2**) shRNAs targeting SPAG9. **(J).** CCK-8 assay of HUVECs treated as in (**I**). **(K).** Transwell migration analysis of HUVECs treated as in (**I**). The migrated and invaded HUVECs were counted at 6 h and 12 h post seeding. Data were shown as mean ± SD. * *P* < 0.05, ** *P* < 0.01, and *** *P* < 0.001, Student's t-test.

To examine the role of SPAG9 in vIRF1-induced malignant transformation, we performed chick chorioallantoic membranes assay (CAMs), which is an *in vivo* model of angiogenesis, following silencing of SPAG9 expression in vIRF1-transduced endothelial cell line with lentivirus-mediated shRNAs (Fig 3A). We found that knockdown of SPAG9 decreased the angiogenic index (Fig 3B and 3C). To further confirm these results, we performed Matrigel plug angiogenesis assay, which is another *in vivo* model of angiogenesis. We revealed that silencing of SPAG9 inhibited the ability of vIRF1 to induce angiogenesis *in vivo* (Fig 3D and 3E). Conversely, overexpression of both SPAG9 and Lef1 enhanced vIRF1-induced angiogenesis (S6 Fig). We investigated the effect of SPAG9 knockdown on vIRF1-induced cell proliferation and migration by using the Cell Count Kit-8 (CCK-8) assay and transwell migration assay, respectively, in vIRF1-transduced HUVECs. We observed that SPAG9 knockdown reduced vIRF1-induced cell proliferation and migration (Fig 3F–3H).

In addition, we transduced KSHV-infected HUVECs with lentivirus-mediated shRNAs targeting SPAG9 and examined the functional consequence on KSHV-induced cell behaviors. We found that knockdown of SPAG9 in KSHV-infected HUVECs decreased cell proliferation and migration (Fig 3I–3K).

### Activation of JNK1/2 signaling by SPAG9 contributes to vIRF1-induced cell proliferation, migration and angiogenesis

**Fig 4:**
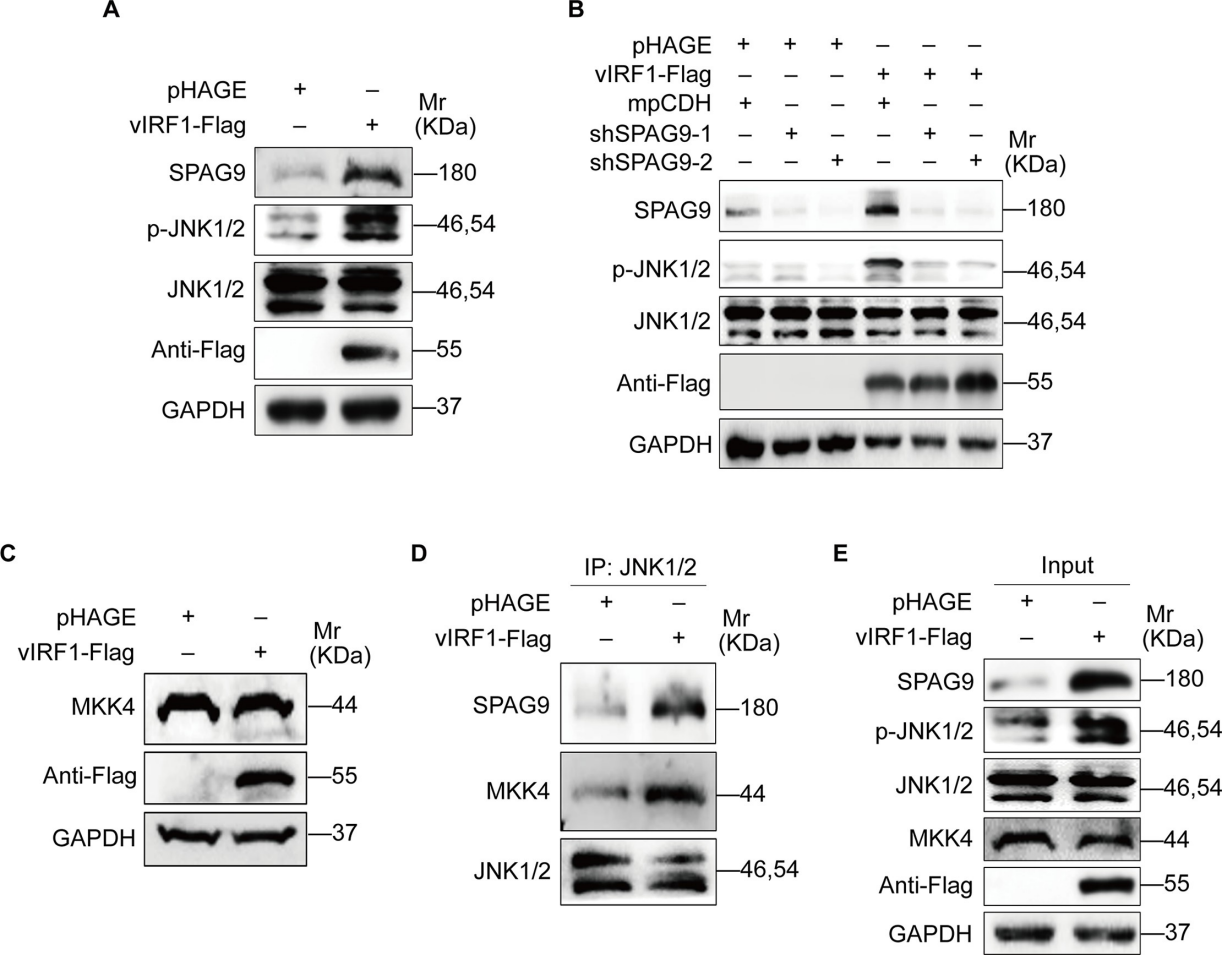
vIRF1-upregulated SPAG9 promotes the binding of MKK4 to JNK1/2 to activate the JNK1/2 signaling. **(A).** Western-blotting analysis of the expression levels of SPAG9, phosphorylated JNK1/2 and total JNK1/2 in vIRF1-expressing HUVECs. **(B).** Western-blotting analysis of phosphorylated JNK1/2 expression in vIRF1-expressing HUVECs transduced with lentivirus-mediated No.1 (shSPAG9-1) and No. 2 (shSPAG9-2) shRNAs targeting SPAG9. **(C).** Western-blotting analysis of MKK4 expression in HUVECs transduced with lentiviral-vIRF1 or its control lentiviral-pHAGE. **(D) and (E).** Immunoprecipitation analyses of the interaction of JNK1/2-SPAG9-MKK4 complex in vIRF1-transduced or its control pHAGE-transduced HUVECs.

SPAG9, as a scaffolding protein, could tether mitogen-activated protein kinase kinases (MKKs) and their targets together to activate JNK signaling pathway [31]. Given the fact that KSHV activates the JNK pathway during primary infection [32], we assumed that SPAG9 might regulate the JNK pathway in vIRF1-induced oncogenic phenotypes. As expected, vIRF1 overexpression activated JNK1/2 (Fig 4A), and knockdown of SPAG9 impaired vIRF1-induced JNK1/2 activation (Fig 4B). Mitogen-activated protein kinase kinase 4 (MKK4) is a well-known upstream kinase of JNK1/2. SPAG9 could recruit MMK4 to JNK1/2 [31]. Western-blotting analysis showed that the level of MKK4 was unchanged in vIRF1 cells (Fig 4C). However, overexpression of vIRF1 increased the amount of JNK1/2-immunoprecipitated SPAG9 and MKK4 (Fig 4D and 4E), indicating that vIRF1-upregulated SPAG9 recruited MKK4 to activate JNK1/2.

**Fig 5:**
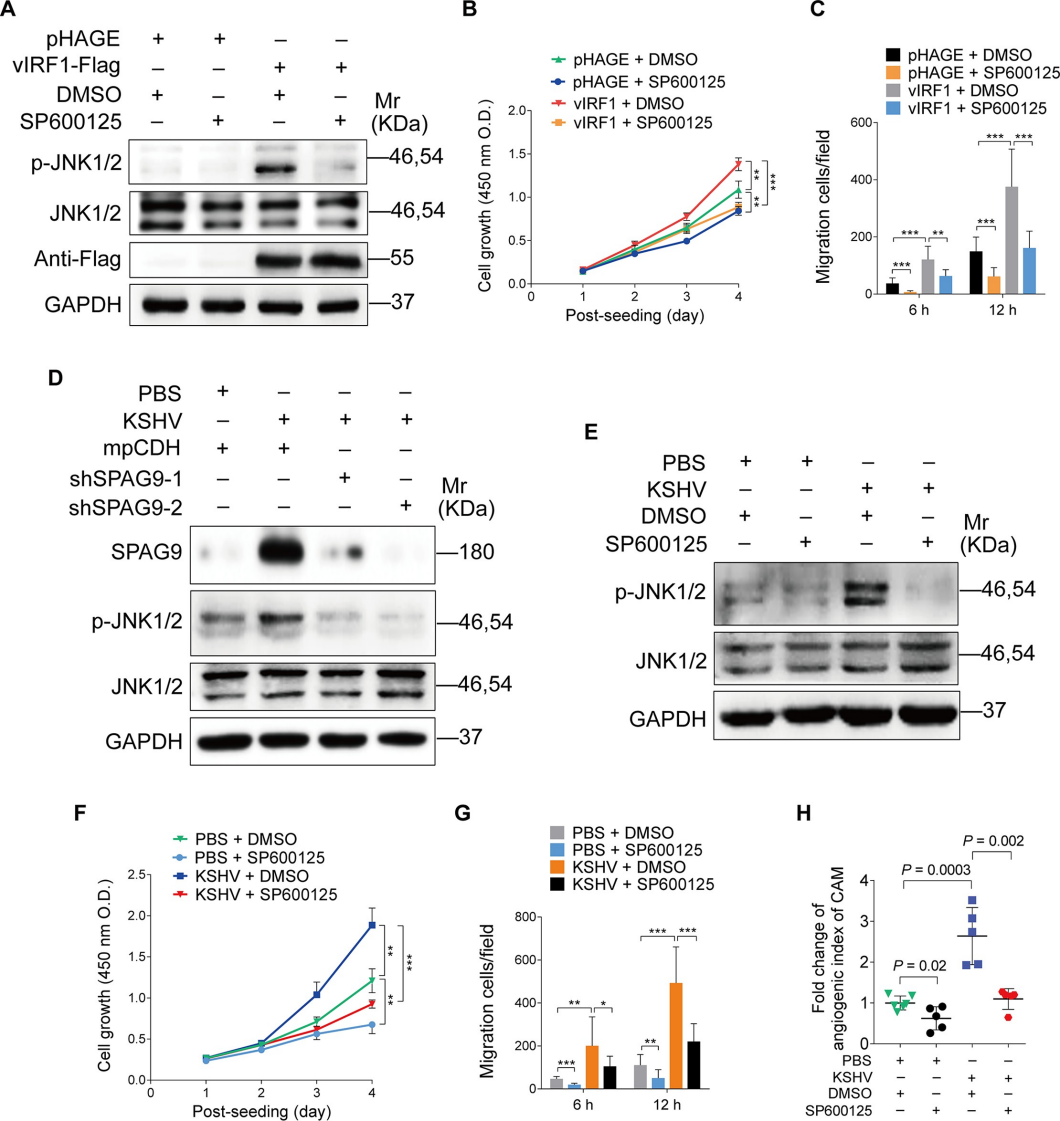
Inhibition of the JNK signaling reduces vIRF1-induced cell proliferation, migration and angiogenesis. **(A).** Western-blotting analysis of phosphorylated JNK1/2 and total JNK1/2 in vIRF1-transduced HUVECs treated with the JNK inhibitor, SP600125 (50 μM) for 48 h. **(B).** CCK-8 assay of HUVECs treated as in (**A**). **(C).** Transwell migration analysis of HUVECs treated as in (**A**). The migrated and invaded HUVECs were counted at 6 h and 12 h post seeding. **(D).** Western-blotting analysis of phosphorylated JNK1/2 and total JNK1/2 expression in KSHV-infected HUVECs transduced with lentivirus-mediated No.1 (shSPAG9-1) and No. 2 (shSPAG9-2) shRNAs targeting SPAG9. **(E).** Western-blotting analysis of phosphorylated JNK1/2 and total JNK1/2 in KSHV-infected HUVECs treated with the JNK inhibitor, SP600125 (50 μM) for 48 h. **(F).** CCK-8 assay of HUVECs treated as in (**E**). **(G).** Transwell migration analysis of HUVECs treated as in (**E**). The migrated and invaded HUVECs were counted at 6 h and 12 h post seeding. **(H).** Lentiviral vIRF1- or its control pHAGE-infected endothelial cell line were treated with the JNK inhibitor, SP600125 (50 μM) for 48 h, and then were subjected to chicken chorioallantoic membranes (CAMs) assay. The quantified results represent mean ± SD. * *P* < 0.05, ** *P* < 0.01, and *** *P* < 0.001, Student's t-test.

Next, we determined whether JNK1/2 signaling mediated vIRF1-induced cell proliferation, migration and angiogenesis. As expected, treatment with SP600125, an inhibitor of JNK pathway, reduced JNK phosphorylation (Fig 5A), as well as cell proliferation and migration induced by vIRF1 (Fig 5B and 5C). Silencing of SPAG9 in KSHV-infected cells also inhibited the activation of JNK1/2 (Fig 5D). Treatment with SP600125 not only decreased the activation of JNK1/2, but also reduced KSHV-induced cell proliferation, migration and angiogenesis (Fig 5E–5H).

Taken together, these results suggested that vIRF1 transcriptional activation of SPAG9 induced JNK1/2 signaling, which promoted angiogenesis, cell proliferation and migration by recruiting MKK4.

### vIRF1 upregulates VEGFA expression by activating the JNK1/2 signaling

**Fig 6:**
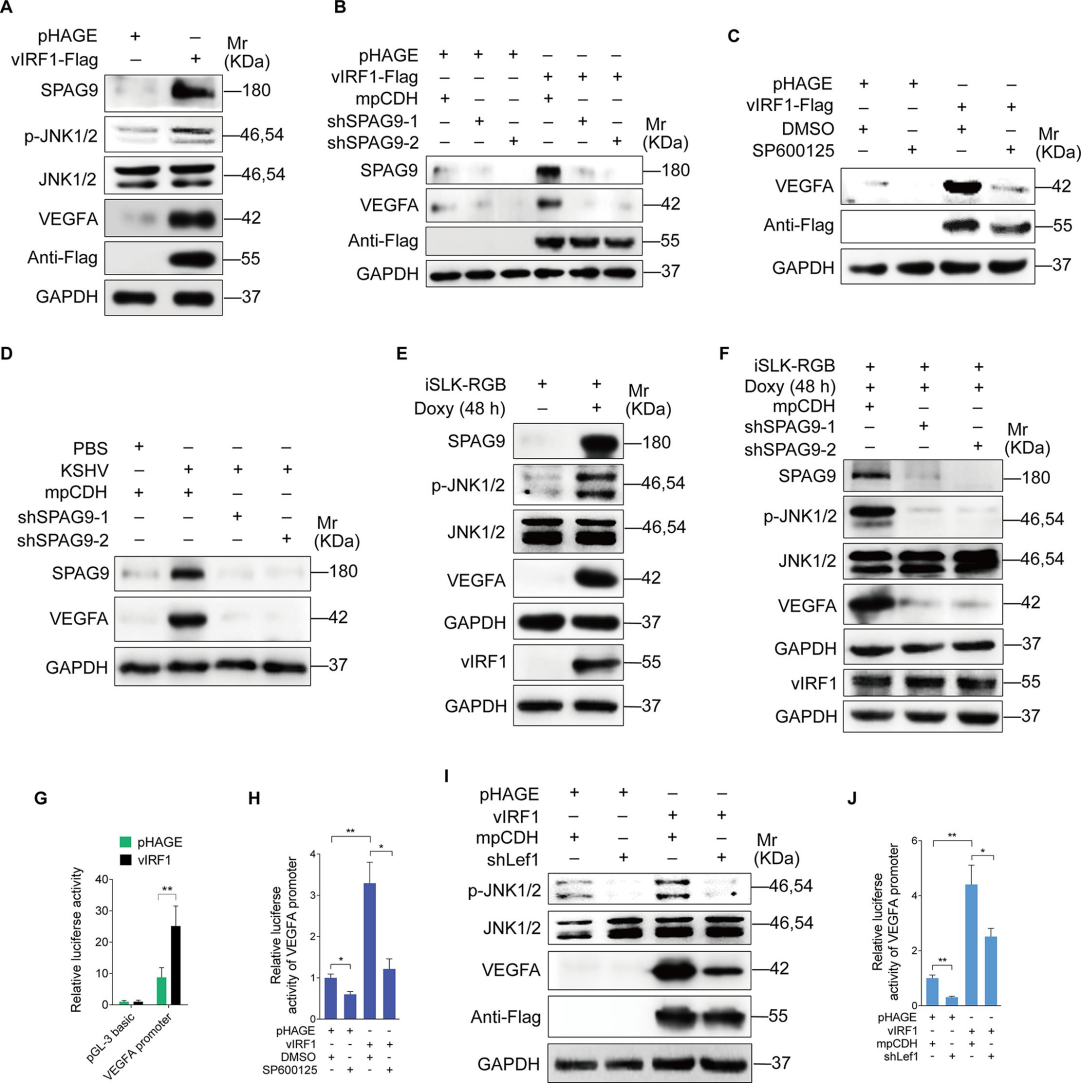
vIRF1 upregulates VEGFA expression by activating the SPAG9/JNK1/2 pathway. **(A).** Western-blotting analysis of SPAG9, phosphorylated JNK1/2, total JNK1/2 and VEGFA expression in HUVECs transduced with lentiviral-vIRF1or its control lentiviral-pHAGE. **(B).** Western-blotting analysis of phosphorylated JNK1/2, total JNK1/2 and VEGFA expression in vIRF1-expressing HUVECs transduced with lentivirus-mediated No.1 (shSPAG9-1) and No. 2 (shSPAG9-2) shRNAs targeting SPAG9. **(C).** Western-blotting analysis of phosphorylated JNK1/2, JNK1/2 and VEGFA in vIRF1-expressing HUVECs treated with the JNK inhibitor, SP600125 (50 μM) for 48 h. **(D).** Western-blotting analysis of phosphorylated JNK1/2, JNK1/2 and VEGFA in KSHV-infected HUVECs treated with the JNK inhibitor, SP600125 (50 μM) for 48 h. **(E).** Western-blotting analysis of SPAG9, phosphorylated JNK1/2, JNK1/2 and VEGFA expression in iSLK-RGB cells treated with doxycycline (Doxy) (1 μg/ml) for 48 h. **(F).** Western-blotting analysis of SPAG9, phosphorylated JNK1/2, JNK1/2 and VEGFA expression in Doxy-induced iSLK-RGB cells transduced with lentivirus-mediated No.1 (shSPAG9-1) and No. 2 (shSPAG9-2) shRNAs targeting SPAG9. **(G).** Luciferase reporter assay of the activity of VEGFA promoter in HUVECs transduced with lentiviral-vIRF1 or its control lentiviral-pHAGE. **(H).** Luciferase reporter assay of the activity of VEGFA promoter in vIRF1-expressing HUVECs treated with the JNK inhibitor, SP600125 (50 μM) for 48 h**. (I).** Western-blotting analysis of phosphorylated JNK1/2, JNK1/2 and VEGFA expression in vIRF1-expressing HUVECs transduced with lentivirus-mediated a mixture of shRNAs targeting Lef1 (shLef1). **(J).** Luciferase reporter assay of the activity of VEGFA promoter in vIRF1-expressing HUVECs transduced with lentivirus-mediated a mixture of shRNAs targeting Lef1 (shLef1). The quantified results represent mean ± SD. * *P* < 0.05, ** *P* < 0.01, and *** *P* < 0.001, Student's t-test.

It is well known that activation of JNK pathway could enhance the binding of phosphorylated c-Jun to the VEGFA promoter to induce VEGFA production [33]. We assumed that VEGFA could be a potential downstream of vIRF1-activated JNK1/2 signaling. Western-blotting analysis showed a much higher level of VEGFA expression in vIRF1-overexpressed cells (Fig 6A). Both loss of SPAG9 and inhibition of JNK1/2 signaling impaired vIRF1-upregulated VEGFA induction (Fig 6B and 6C). Knockdown of SPAG9 also diminished the production of VEGFA induced by KSHV (Fig 6D). Similarly, robust phosphorylated JNK1/2 and VEGFA were observed following KSHV reactivation in iSLK-RGB cells by treatment with doxycycline (Fig 6E), and this induction was successfully blocked by silencing SPAG9 (Fig 6F). A decrease of VEGFA expression level was observed in iSLK-RGB-K9 mutant cells compared to iSLK-RGB cells in reactivated KSHV-infected cells (S7 Fig). In a luciferase reporter assay, vIRF1 upregulated VEGFA promoter activity (Fig 6G), and inhibition of JNK1/2 pathway blocked this upregulated ability (Fig 6H). We also observed the reduction of phosphorylated JNK1/2 and VEGFA expression (Fig 6I), as well as VEGFA promoter activity (Fig 6J) following knockdown of Lef1. In addition, knockdown of Lef1 in KSHV-infected cells not only inhibited the activation of JNK1/2 and expression of VEGFA (S8A Fig), but also impaired KSHV-induced cell proliferation, migration and angiogenesis (S8B–S8D Fig).

Together these data suggested that by hijacking Lef1, vIRF1 transcriptionally activated SPAG9 to promote angiogenesis, cell proliferation and migration via activation of the JNK/VEGFA pathway.

### vIRF1 plays an important role in KSHV-induced cellular transformation and angiogenesis by regulating the SPAG9/JNK/VEGFA axis

**Fig 7:**
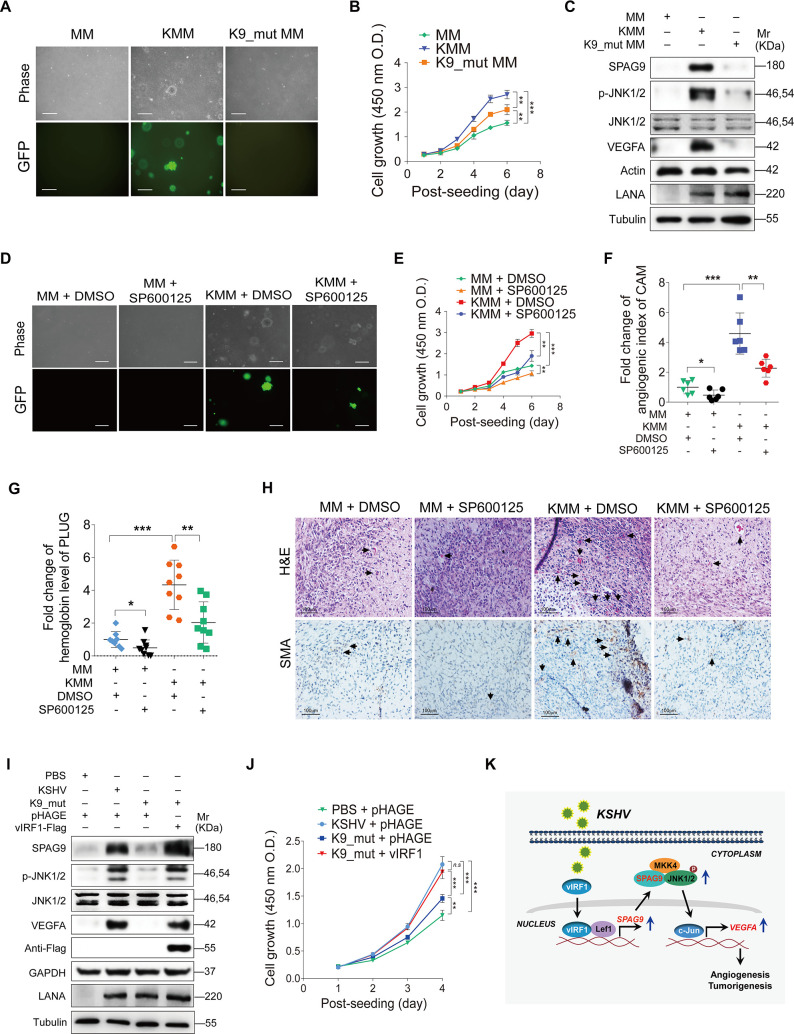
vIRF1 induces cellular transformation and angiogenesis by activating the SPAG9/JNK/VEGFA pathway. **(A).** Soft agar assay of MM cells, KSHV-infected and transformed MM cells (KMM) and a mutant with a deletion of KSHV ORF-K9 infected MM cells (**K9_Mut**) (MOI of 3). The representative images were captured at 2 weeks post seeding. Magnification, ×100. Scar bars, 40 μm. **(B).** CCK-8 assay of cells treated as in (**A**). **(C).** Western-blotting analysis of SPAG9, phosphorylated JNK1/2, JNK1/2 and VEGFA expression in cells treated as in (**A**). **(D).** Soft agar assay of MM and KMM cells treated with the JNK inhibitor, SP600125 (50 μM) for 48 h**.** The representative images were captured at 2 weeks post seeding. Magnification, ×100. Scar bars, 40 μm. **(E).** CCK-8 assay of cells treated as in (**D**). **(F).** Chicken chorioallantoic membranes (CAMs) assay of cells treated as in (**D**). **(G).** Matrigel plug assay in mice of cells treated as in (**D**). **(H).** Hematoxylin and eosin (H&E) staining analysis of histologic features (up; ×400) and immunohistochemical (IHC) staining analysis of the expression of SMA (down; ×400) in plugs in mice induced by cells treated as in (**D**). The newly formed blood vessels and the SMA expression were pointed out by black arrows, respectively. **(I).** Western-blotting analysis of SPAG9, phosphorylated JNK1/2, JNK1/2 and VEGFA expression in HUVECs treated with PBS (**PBS**) or infected with wild-type KSHV (**KSHV_WT**) (MOI of 3) or vIRF1 mutant virus (**K9_mut**) (MOI of 3) followed by transduction with lentiviral vIRF1 at MOI 2 at 6 hpi. **(J).** CCK-8 assay of cells treated as in (**I**). **(K).** A schematic working model of the mechanism by which vIRF1 facilitates angiogenesis and cell transformation. vIRF1 enhanced SPAG9 transcription by interacting with Lef1 to promote the transcriptional activity of Lef1. Increased SPAG9 expression enhanced the activation of JNK1/2 pathway and VEGFA transcription contributing to KSHV-induced angiogenesis and tumorigenesis.

To determine the role of vIRF1 in cellular transformation and angiogenesis, we examined cellular transformation of a KSHV mutant virus with deleted ORF-K9 [29] in rat primary embryonic metanephric mesenchymal (MM) cells [34]. Soft agar assay showed that deletion of vIRF1 from KSHV genome abolished the ability of KSHV-infected cells to form colonies (Fig 7A). Cells infected by the K9_mut virus also showed a lowered cell proliferation rate (Fig 7B). Western-blotting confirmed that deletion of vIRF1 decreased expression levels of SPAG9, phosphorylated-JNK1/2 and VEGFA when compared to those of KSHV_WT virus-infected cells (Fig 7C). To further determine the role of Lef1/SPAG9 in KSHV-induced cell proliferation, we knockdown Lef1 and SPAG9, respectively, in KMM cells, and found that loss of Lef1 or SPAG9 impaired KSHV-induced cell proliferation (S9A–S9D Fig).

To examine the role of the JNK1/2 pathway in KSHV-induced cellular transformation, we treated KSHV-infected cells with SP600125, and found that inhibition of JNK activation not only reduced KSHV-induced efficiency of colony formation (Fig 7D) and cell proliferation (Fig 7E), but also decreased KSHV-induced angiogenesis (Fig 7F and 7G). We performed hematoxylin and eosin staining and immunohistochemistry to observe the newly formed blood vessels and the levels of smooth muscle actin (SMA), which is a proangiogenic factor, in the mice plugs. As shown in Fig 7H, there were reduced neovascularization and less SMA-positive cells in the SP600125-treated plugs of KMM cells than the untreated control cells. Similarly, HUVECs infected with K9_mut virus had reduced levels of SPAG9, phosphorylated JNKs and VEGFA expression compared to those infected by KSHV_WT virus (Fig 7I). vIRF1 complementation in cells infected by K9_mut virus completely reversed the effect (Fig 7I). Importantly, loss of vIRF1 lowered KSHV-induced endothelial cell proliferation while complementation with vIRF1 was sufficient to rescue KSHV-induced the proliferation (Fig 7J). Collectively, these data indicated that vIRF1 mediated KSHV-induced cellular transformation and angiogenesis by enhancing the SPAG9/JNK/VEGFA axis.

## Discussion

KSHV vIRF1 was characterized as a lytic protein and its expression can be induced by 12-*O*-tetradecanoyl-phorbol-13-acetate (TPA) and sodium butyrate (NaB) in KSHV-positive primary effusion lymphoma (PEL) cells [16, 35]. However, low levels of vIRF1 expression were detectable in latently KSHV-infected KS tumors and PEL cells [36–38]. Hence, vIRF1 expression in tumor tissue could have occurred in a subset of lytically infected cells. In fact, about 1–3% of the KSHV-infected cells in KS tumors express viral lytic proteins [39–42]. The low rate of spontaneous lytic replication is presumed to produce infectious virions to infect new cells. At the same time, during lytic replication and *de novo* infection, virus-encoded cytokines and host-induced cytokines contribute to KS pathogenesis through autocrine and paracrine mechanisms [43]. Importantly, our most recent study reported that vIRF1-positive cells could be detected in a small number of KSHV-infected cells in KS lesions, indicating that not all KSHV-infected cells had detectable vIRF1 protein and the expression of vIRF1 protein is most likely due to viral lytic replication [30]. In this study, we have shown that SPAG9-positive cells were more than LANA-positive cells in KS tissues, suggesting that, besides vIRF1 transcriptional activation of SPAG9, a paracrine mechanism might also be involved in upregulation of SPAG9 expression by soluble factors.

As the first identified oncogenic protein encoded by KSHV, vIRF1 has been reported to transform mouse embryonic fibroblasts (NIH3T3) cells [44]; however, its role in KSHV-induced tumors and its underlying mechanism remains largely unknown. Our recent work has shown that vIRF1 promotes cell migration, invasion and proliferation [29]. Here, we have further demonstrated that, besides cell motility, vIRF1 promotes angiogenesis and cellular transformation. These findings reveal a novel and pivotal role of vIRF1 in the pathogenesis of KSHV-induced malignancies.

Human tumor antigens are potential targets for tumor immunotherapy. Cancer/testis (CT) antigens, a unique group of antigens, could induce spontaneous immune responses in cancer patients and have been proposed to be potential cancer vaccine targets [45]. Sperm associated antigen 9 (SPAG9), as a novel member of the CT antigen family, was first identified from a human testis cDNA library in 1998 [46], and later found to be aberrantly activated and highly expressed in numerous types of human cancer. SPAG9 contains c-Jun N-terminal kinase (JNK)-binding domain [47], and it is the latest member of the family of JNK-interacting proteins, also called JNK-interacting leucine zipper protein (JLP) [47]. Evidence suggest that SPAG9 participates in multiple pathophysiological process through activating MAPK signaling by tethering both JNK and p38MAPK. In this study, we have observed that SPAG9 is expressed at a higher level in vIRF1-transduced endothelial cells, KSHV-infected endothelial cells, and KS tissues. Mechanistically, vIRF1 functions as a transcription cofactor to promote the binding of transcription factor Lef1 to the promoter of *SPAG9*, resulting in enhancement of SPAG9 transcription and expression. Silencing of SPAG9 inhibited vIRF1-induced angiogenesis, cell proliferation, and migration, indicating the importance of SPAG9 in the development of KS. Interestingly, a higher antibody response against SPAG9 was observed in cancer patients [22], suggesting its therapeutic potential as a tumor immunotherapy target. Therefore, it would be interesting to examine the antibody level against SPAG9 in KS patients and determine whether targeting SPAG9 could be a valid immunotherapy strategy for KSHV-associated tumors.

JNK activation leads to phosphorylation of downstream molecules, which is associated with a wide spectrum of cellular processes, including cell death/survival, inflammation, cell differentiation, and cell proliferation [48]. JNK activation was initially identified as a tumor suppressor, however, more and more evidences suggest that JNK hyperactivation is beneficial for malignant transformation and tumor growth, as well as drug resistance [49, 50]. With regard to KSHV infection and JNK signaling, it has been reported that KSHV activates the JNK pathway during primary infection in a productive infection system of human umbilical vein endothelial cells (HUVECs), and this pathway modulates the early viral entry events and subsequent viral lytic replication [32, 51]. Inhibition of JNK blocks KSHV lytic replication at the early stage(s) of reactivation in TPA-induced PEL cells [52]. These observations suggest the important role of JNK pathway in KS pathogenesis. JNK is activated by upstream MAPK kinases (MKKs), including MKK4 and MKK7 [53]. SPAG9 could be associated with MKK4, and involved in bringing MKK4 and JNK together [31]. In agreement with this finding, here we show that vIRF1 stimulates JNK1/2 signaling by enhancing the transcription and expression of SPAG9. Upregulated SAPG9 activates JNK1/2 by recruiting more MKK4 to JNK1/2, resulting in increased JNK1/2 phosphorylation. Furthermore, blocking the JNK1/2 signaling impairs KSHV-induced cellular transformation, angiogenesis, and cell invasion, indicating that the JNK1/2 pathway may be a potential therapeutic target for KSHV-induced tumors. However, we observed a higher level of p-JNK1/2 in KSHV-infected cells than in vIRF1-transduced cells in the current study. This is likely due to the fact that other KSHV proteins and miRNAs might also induce JNK activation. These include miR-K6-3p [54], miR-K3 [55], viral FLICE-inhibitory protein (vFLIP) [56], viral interleukin-6 (vIL-6) [57], and ORF-K15 [58]. In agreement with these results, KSHV infection induces faster cell proliferation and cell migration than vIRF1 does.

VEGFA is a well-known angiogenic factor important for vascular permeability, and survival of newly-formed vasculature [59]. VEGFA, which is expressed in the spindle cells of KS lesions, is synergistically induced by inflammatory cytokines [60]. A higher level of VEGFA was observed in KSHV-positive PEL cell lines compared to uninfected cells [61]. More excitingly, endothelial cells treated with conditioned medium collected from these angiogenic cell lines promoted the formation of the capillary network [62]. KSHV infection of endothelial cells had a higher level of VEGFA expression compared to uninfected cells [63]. Many KSHV-encoded products have been shown to induce VEGFA expression. For instance, KSHV vIL-6 induced VEGFA expression, which promoted vascular permeability [64] while K1 promoted the expression and release of VEGFA [65]. Furthermore, KSHV miRNAs also increased the expression of VEGFA, as well as the secretion of VEGFA protein [66, 67]. It is well known that activation of JNK signaling induces VEGFA production [33, 68]. Here, we demonstrated that vIRF1 promoted VEGFA transcription by activating the SPAG9/JNK1/2 pathway. These novel findings revealed an important mechanism of VEGFA induction by KSHV, and further supported the essential role of VEGFA in KSHV-induced angiogenesis and cellular transformation.

In conclusion, we have found that KSHV vIRF1 upregulates SPAG9, which is essential for vIRF1-induced cellular transformation, angiogenesis and cell invasion. vIRF1 enhances SPAG9 transcription by interacting with Lef1 to promote the transcriptional activity of Lef1. Increased SPAG9 expression enhances the activation of JNK1/2 pathway and VEGFA transcription, contributing to KSHV-induced angiogenesis and tumorigenesis (Fig 7K). Our findings illustrate a novel mechanism of vIRF1-induced cellular transformation, angiogenesis and cell invasion, and demonstrate a vital role of vIRF1, and its regulatory proteins and pathways in the pathogenesis of KSHV-associated malignancies, thus reveal potential therapeutic targets for KS.

## Materials and methods

### Ethics statement

The Institutional Ethics Committee of the First Affiliated Hospital of Nanjing Medical University reviewed and ethically approved the clinical section of the research (Nanjing, China; Study protocol # 2015-SR-116). Written informed consent was obtained from all participants, and all samples were anonymized. All participants were adults. Embryonated chicken eggs were purchased from Qian Yuan Hao Biological Co., LTD. Nanjing Biological Pharmaceutical Factory (Nanjing, China), and were housed under SPF conditions. Male athymic BALB/c nu/nu mice of four-week-old were from Nanjing Biomedical Research Institute of Nanjing University (Nanjing, China) and were maintained under pathogen-free conditions. All animal care and use protocols were performed in accordance with Laboratory Animal Management Regulations approved by the State Council of People’s Republic of China. The Institutional Animal Care and Use Committee of Nanjing Medical University approved the animal experiments (Animal protocol # NJMU/IACUC_2013-8-18-01).

### Cell culture and plasmids

The iSLK-RGB-BAC16 and iSLK-RGB-K9 mutant cells were established and cultivated in DMEM supplemented with 10% fetal bovine serum (FBS), 1 μg/ml puromycin, 250 μg/ml G418, and 1.2 mg/ml hygromycin B [69]. Rat primary embryonic metanephric mesenchymal (MM) cells and KSHV-transformed MM cells (KMM) were maintained as previously described [34]. Human embryonic kidney HEK293T (ATCC, CRL-11268) were maintained as previously described [70]. A human umbilical vein endothelial cell line (catalog #CRL-2922TM; ATCC, Manassas, VA, USA) used for chicken chorioallantoic membranes (CAMs) assay and matrigel plug assay were cultured in DMEM supplemented with 10% fetal bovine serum (FBS). Primary human umbilical vein endothelial cells (HUVECs), which were used for all assays except for luciferase report assay, were isolated from the interior of the umbilical vein of human umbilical cords by digestion with collagenase (Sigma, St. Louis, MO, USA) as previously delineated [71]. HUVECs were cultured in complete EBM-2 culture media (LONZA, Allendale, NJ, USA) and used between passage 3 and 6. Cells and cell lines used in this study were examined for mycoplasma contamination using Myco-Blue Mycoplasma Detector (D103-01/02, Vazyme Biotech Co., Ltd, Nanjing, China).

Flag-vIRF1 was cloned by inserting the coding sequences into plasmid pHAGE-CMV-MCSIzsGreen as previously described [70, 72]. A 2000 bp fragment of SPAG9 (-1500 to +500) promoter was amplified and subcloned into pGL3-Basic vector containing the firefly luciferase reporter gene (Promega, Madison, WI, USA). Short hairpin RNAs (shRNAs) were constructed as previously described [29, 55]. The target sequences of shSPAG9 (for human) are 5’-GCTTCTCCAGTGATGGATA-3’ (shSPAG9-1) and 5’-GCAATGACTCAGATGCATA-3’ (shSPAG9-2). The target sequences of shSPAG9 (for Rat) are 5’- AGCTGAAGATGCAAGGC-3’ and 5’-GGACGTTTCTGCTCTGG -3’. The target sequences of shLef1 (for Rat) are 5’- GCGACCTAATGCACGTG -3’ and 5’- GCAAGAAGAAGAAGAGG -3’. The control vector of all the shRNAs was a modified lentivirus pCDH plasmid (mpCDH for short), which contains both GFP and RFP cassettes, and was generated in our previous study [55].

### Transfection, reagents, antibodies, and western-blotting

Transfection of HEK293T cells were performed with the Lipofectamine 2000 Reagent (Invitrogen, Carlsbad, CA, USA). Transfection of HUVECs was performed with the Effectence transfection reagent (Qiagen, Suzhou, Jiangsu, China). SP600125 were purchased from Selleck Chemicals (Shanghai, China). Antibodies used were anti-SPAG9 rabbit antibody from Abcam (Cambridge, MA, USA), anti-p-JNK rabbit antibody and anti-JNK rabbit antibody from Beyotime Institute of Biotechnology (Nantong, Jiangsu, China), anti-Flag rabbit antibody from Cell Signaling Technologies (Beijing, China), anti-α-Tubulin mouse antibody, anti-GAPDH mouse antibody, anti-Actin mouse antibody, and anti-VEGFA rabbit antibody from Santa Cruz Biotechnology (Dallas, TX, USA). The polyclonal rabbit anti-vIRF1 antibodies were kindly provided by Dr. Gary Hayward from Viral Oncology Program, The Johns Hopkins School of Medicine.

### Cell proliferation and transwell migration assays

Cell Count Kit-8 (Dojindo Molecular Technologies, Tokyo, Japan) was used for Cell proliferation assay as previously described [73]. Transwell chambers (8 μm) from Merck Millipore (Darmstadt, Germany) was adopted for transwell migration as previously described [55, 74]. HUVECs (1 × 10^5^) were seeded into chambers, and the chambers were harvested, fixed and stained after 6 h or 12 h incubation. The migrated cells were photographed and calculated by counting stained cells in a double-blinded manner by two observers.

### Soft agar assay

Soft agar assay was performed as previously described [75]. Briefly, cells (2 × 10^4^) were suspended in 0.4% top agar (BD Biosciences) and then plated onto 0.8% base agar in six well-plates. The plates were incubated at 37°C with 5% CO_2_ for 2 weeks. Random fields were chosen and photographed. Colony areas were counted by NIH Image J software. The colony sizes of 20 μm or larger were scored for calculation of the percentage of soft agar colony.

### Luciferase reporter assay

Promega dual-luciferase reporter assay system were adopted for luciferase reporter assay according to the manufacturer’s protocol as previously described [54]. The renilla vector pRL-TK was used to normalize transfection efficiency.

### Real-time PCR analysis of mRNA transcripts

Total RNA was extracted using RNA Isolator Total RNA Extraction Reagent (Vazyme Biotech Co., Ltd, Nanjing, China) according to the manufacturer’s instructions. Reverse transcription was performed by HiScript Q RT SuperMix (Vazyme Biotech Co., Ltd, Nanjing, China). Quantitative PCR (qPCR) analysis was performed by AceQ qPCR SYBR Green Master Mix (Vazyme Biotech Co., Ltd, Nanjing, China). The sequences of the primers for PCR are as follows: 5’-GAAGGTGAAGGTCGGAGTC-3’ (forward) and 5’-GAAGATGGTGATGGGATTTCC-3’ (reverse) for GAPDH; 5’-CAAGGCGGATCTAAAGCTACC-3’ (forward) and 5’- TTGGCGCATCTGTAACCTTCA -3’ (reverse) for SPAG9.

### Chicken chorioallantoic membranes (CAMs) assay

CAMs were performed with 9-day-old embryos as previously described [73]. At least 5 chicken embryos were used for each treatment. Embryos were randomly divided. Briefly, fertilized White Leghorn chicken eggs were incubated at 37°C under conditions of constant humidity. The developing CAMs were separated from the shell by opening a window at the broad end of the egg above the air sac on day 9. The opening was sealed and the eggs were returned to the incubator. To study cellular angiogenesis, the cells were suspended in a medium containing 50% High Concentration Matrigel (BD Biosciences). Aliquots (30 μl) of the mixture were then applied onto the CAMs of 9-day old embryos. The area around the implanted Matrigel was photographed 4–5 days after implantation, and the blood vessels were counted by two observers in a double-blind manner.

### Matrigel plug assay

For Matrigel Plug Assay, four-week-old male *nu/nu* nude mice were used. The plug assay was performed as previously described [74]. All procedures were performed in accordance with the policies of Nanjing Medical University Experimental Animal Welfare Ethics Committee. At least 6 mice were used in each treatment. Nude mice were randomly divided. Mixed 5 x 10^6^ cells with the high concentration Matrigel (BD Biosciences, Bedford, MA, USA), and then injected into the right flanks of nude mice. 10 days later, harvest and photograph the plugs using stereomicroscope. Drabkin’s reagent kit (Sigma-Aldrich) was used for the measurement of the hemoglobin content. Nude mice were randomly divided and no statistical method was used to predetermine sample sizes.

### Chromatin Immunoprecipitation (ChIP) assay

EZ-Magna ChIP A/G Chromatin Immunoprecipitation Kit (Merck, Darmstadt, Germany) was used for ChIP analysis as previously described [76]. Cells (10^7^) cross-linked by 1% formaldehyde were harvested to suffer sonication. Precipitate obtained DNA fragments with indicated antibodies. Specific primers were used for RT-qPCR. The sequences of SPAG9 promoter primers are as follows: 5’- TTTCCCGTTGTAGCTGCGT -3’ and 5’- GTGACGAGTTAACTTAGCTGG’ for site (1); 5’- TCCGGGGCCGTGATGTCGGAG -3’ and 5’- AGCTCCAGCTCCACCTGGT’ for site (2).

### Co-Immunoprecipitation (Co-IP)

Specific antibodies were used for immunoprecipitations using a standard protocol as previously described [54]. Briefly, Co-IP assay was executed according to the standard protocol for detecting protein–protein interactions. The IP lysis/wash buffer containing protein inhibitor cocktail was employed to collect protein lysates. To identify binding proteins, the beads were washed and eluted for Western blotting. The secondary antibodies from IPKine HRP Goat Anti-Mouse or Anti-Rabbit IgG LCS (Abbkine Scientific Co., Ltd. Wuhan, China) were used, which could avoid the detection of the heavy chains of IgG by specifically reacting with kappa light chains on IgG.

### Immunohistochemistry (IHC)

IHC was carried out as previously described [55, 74]. The antibodies used for IHC are as follows: anti-KSHV LANA (Advanced Biotechnologies Inc., Columbia, MD, USA), anti-SPAG9 (Abcam, Cambridge, MA, USA), anti-rabbit immunoglobulin G (IgG) (Beyotime Institute of Biotechnology, Nantong, Jiangsu, China), and anti-smooth muscle actin (SMA) rabbit polyantibody (Abbiotec, San Diego, CA, USA). Secondary antibodies used in this study were horseradish peroxidase (HRP)-labeled goat anti-rat or anti-rabbit from Beyotime Institute of Biotechnology (Nantong, Jiangsu, China). DAB (3,3'-diaminobenzidine) Peroxidase (HRP) Substrate Kit (VECTOR LABORATORIES, INC., Burlingame, USA) was used to visualize staining.

### Statistical analysis

Results are presented as the means ± SD. Statistical analysis was on account of Student’s *t*-test. *P* values of less than 0.05 was considered significant. The experiments were not randomized, and investigators were not blinded to allocation during experiments and outcome assessment. All the experiments were repeated at least three times, unless otherwise stated.

## Supporting information

S1 Fig Immunohistochemical staining of KS lesion and normal skin. Immunohistochemical staining of isotype control immunoglobulin G (IgG) in normal skin, and skin KS of patient #1 (**Skin KS1**). Magnification, ×200.
(TIF)

S2 Fig The mRNA levels of vIRF1 and SPAG9 at the different time points during KSHV infection. **(A).** RT-qPCR analysis of mRNA level of **vIRF1** in HUVECs infected by KSHV wild-type virus (**KSHV**) (MOI of 3). Samples were collected at 0 h, 1 h, 6 h, 12 h, 24 h, 48 h, and 72 h after KSHV infection. Undet., undetermined. **(B).** RT-qPCR analysis of mRNA level of **SPAG9** in HUVECs infected by KSHV wild-type virus (**KSHV**) (MOI of 3). Samples were collected at 0 h, 1 h, 6 h, 12 h, 24 h, 48 h, and 72 h after KSHV infection. **(C).** Lack of vIRF1 reduces SPGA9 mRNA transcript induced by KSHV. RT-qPCR analysis of mRNA level of SPAG9 in HUVECs treated with PBS (**PBS**) or infected with wild-type KSHV (**KSHV_WT**) (MOI of 3) or vIRF1 mutant virus (**K9_mut**) (MOI of 3) for 30 h. Data were shown as mean ± SD. *** *P* < 0.001, Student's t-test. *n*.*s*, not significant.
(TIF)

S3 Fig vIRF1 increases the transcription activity of SPAG9 promoter in a dose-dependent manner. **(A).** Western-blotting analysis of vIRF1 (**Anti-Flag**) in HUVECs transduced with different MOIs (1, 2 and 4) of lentiviral-vIRF1 or its control lentiviral-pHAGE. **(B).** Luciferase reporter assay of the activity of SPAG9 promoter in HUVECs transduced with different MOIs (1, 2 and 4) of lentiviral-vIRF1 or its control lentiviral-pHAGE. Data were shown as mean ± SD. * *P* < 0.05 and *** *P* < 0.001, Student's t-test.
(TIF)

S4 Fig Lack of vIRF1 impairs the transcription activity of SPAG9 promoter induced by KSHV. Luciferase reporter assay of the activity of SPAG9 promoter in HUVECs treated with PBS (**PBS**) or infected with wild-type KSHV (**KSHV_WT**) (MOI of 3) or vIRF1 mutant virus (**K9_mut**) (MOI of 3) for 30 h. Data were shown as mean ± SD. ** *P* < 0.01 and *** *P* < 0.001, Student's t-test.
(TIF)

S5 Fig Knockdown of Lef1 in KSHV-infected cells reduces both mRNA and protein levels of SPAG9. **(A).** RT-qPCR analysis of mRNA level of **SPAG9** expression in KSHV-infected HUVECs transduced with a mixture of lentivirus-mediated shRNAs targeting Lef1 (**shLef1**). Data were shown as mean ± SD. ** *P* < 0.01 and *** *P* < 0.001, Student's t-test. **(B).** Western-blotting analysis of SPAG9 expression in KSHV-infected HUVECs transduced with a mixture of lentivirus-mediated shRNAs targeting Lef1 (**shLef1**).
(TIF)

S6 Fig Overexpression of SPAG9 and Lef1 increases vIRF1-induced angiogenesis. Lentiviral vIRF1- or its control pHAGE-infected endothelial cell line were transduced with lentivirus-SPAG9 (SPAG9), lentivirus-Lef1 (Lef1) or its control pHAGE (pHAGE), respectively, and then were subjected to chicken chorioallantoic membranes (CAMs) assay. Quantification of CAMs assay was showed. Data were shown as mean ± SD. * *P* < 0.05 and ** *P* < 0.01, Student's t-test.
(TIF)

S7 Fig vIRF1 up-regulates the VEGFA expression during KSHV reactivation. Western-blotting analysis of VEGFA expression in iSLK-RGB cells and iSLK-RGB-K9 mutant cells treated with doxycycline (Doxy) (1 μg/ml) for 48 h.
(TIF)

S8 Fig Knockdown of Lef1 impaired KSHV-induced cell proliferation, migration and angiogenesis. **(A).** Western-blotting analysis of the expressions of SPAG9, p-JNK1/2, and VEGFA in KSHV-infected HUVECs transduced with a mixture of lentivirus-mediated shRNAs targeting Lef1 (**shLef1**). **(B).** CCK-8 assay of HUVECs treated as in (**A**). **(C).** Transwell migration analysis of HUVECs treated as in (**A**). The migrated HUVECs were counted at 6 h and 12 h post seeding. **(D).** PBS-treated or KSHV-infected endothelial cell line were transduced with a mixture of lentivirus-mediated shRNAs targeting Lef1 (**shLef1**) for 48 h, and then were subjected to chicken chorioallantoic membranes (CAMs) assay. Data were shown as mean ± SD. * *P* < 0.05, ** *P* < 0.01 and *** *P* < 0.001, Student's t-test.
(TIF)

S9 Fig Knockdown of both Lef1 and SPAG9 expression reduces KSHV-induced activation of JNK1/2, VEGFA expression and cell proliferation. **(A).** Western-blotting analysis of Lef1, SPAG9, p-JNK1/2 and VEGFA expression in MM and KMM cells transduced with a mixture of lentivirus-mediated shRNAs targeting Lef1 (**shLef1**). **(B).** CCK-8 assay of cells treated as in (**A**). **(C).** Western-blotting analysis of SPAG9, p-JNK1/2 and VEGFA expression in MM and KMM cells transduced with a mixture of lentivirus-mediated shRNAs targeting SPAG9 (**shSPAG9**). **(D).** CCK-8 assay of cells treated as in (**C**). Data were shown as mean ± SD. ** *P* < 0.01 and *** *P* < 0.001, Student's t-test.
(TIF)
