# Correction: Sperm associated antigen 9 promotes oncogenic KSHV-encoded interferon regulatory factor-induced cellular transformation and angiogenesis by activating the JNK/VEGFA pathway

**DOI:** 10.1371/journal.ppat.1010232

**Published:** 2022-01-07

**Authors:** Wan Li, Fei Wang, Jiale Shi, Qi Feng, Yuheng Chen, Xiaoyu Qi, Cong Wang, Hongmei Lu, Zhongmou Lu, Xuemei Jia, Qin Yan, Shou-Jiang Gao, Chun Lu

In Fig 1D, the incorrect image is included for the immunohistochemical (IHC) staining of LANA in normal skin. The interpretation and the conclusions of the paper are unchanged. Please see the correct Fig 1 here.

**Fig 1:**
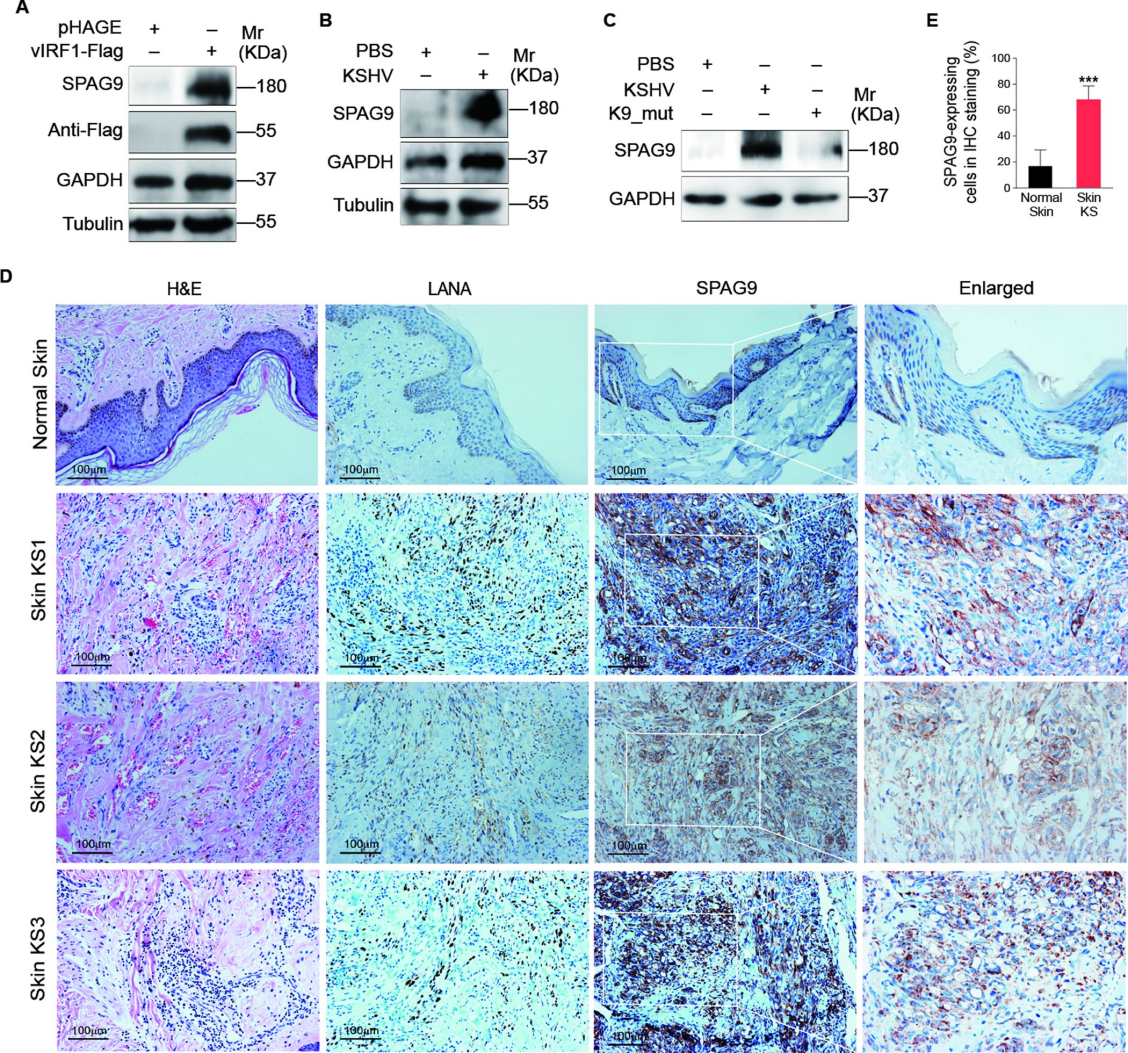
SPAG9 is upregulated in vIRF1-transduced HUVECs and KSHV-infected HUVECs. (A). Western-blotting analysis of SPAG9 in HUVECs transduced with lentiviral-vIRF1 or its control lentiviral-pHAGE (MOI of 2). (B). Western-blotting analysis of SPAG9 in HUVECs treated with PBS (PBS) or infected by KSHV wild-type virus (KSHV) (MOI of 3). (C). Western-blotting analysis of SPAG9 expression in HUVECs treated with PBS (PBS) or infected with wild-type KSHV (KSHV_WT) (MOI of 3) or vIRF1 mutant virus (K9_mut) (MOI of 3) for 30 h. (D). Hematoxylin and eosin (H&E) staining and immunohistochemical staining (IHC) of KSHV LANA, SPAG9 in normal skin, skin KS of patient #1 (Skin KS1), patient #2 (Skin KS2), and patient #3 (Skin KS3). Magnification, ×200, ×400. (E). Results were quantified in (D). Data were shown as mean ± SD. *** P < 0.001, Student’s t-test.
