# Supplementary figures and images for: Sperm associated antigen 9 promotes oncogenic KSHV-encoded interferon regulatory factor-induced cellular transformation and angiogenesis by activating the JNK/VEGFA pathway
Source: PLoS Pathog. 2020 Aug 10;16(8):e1008730. doi: 10.1371/journal.ppat.1008730 (PMC7446834; doi:10.1371/journal.ppat.1008730)

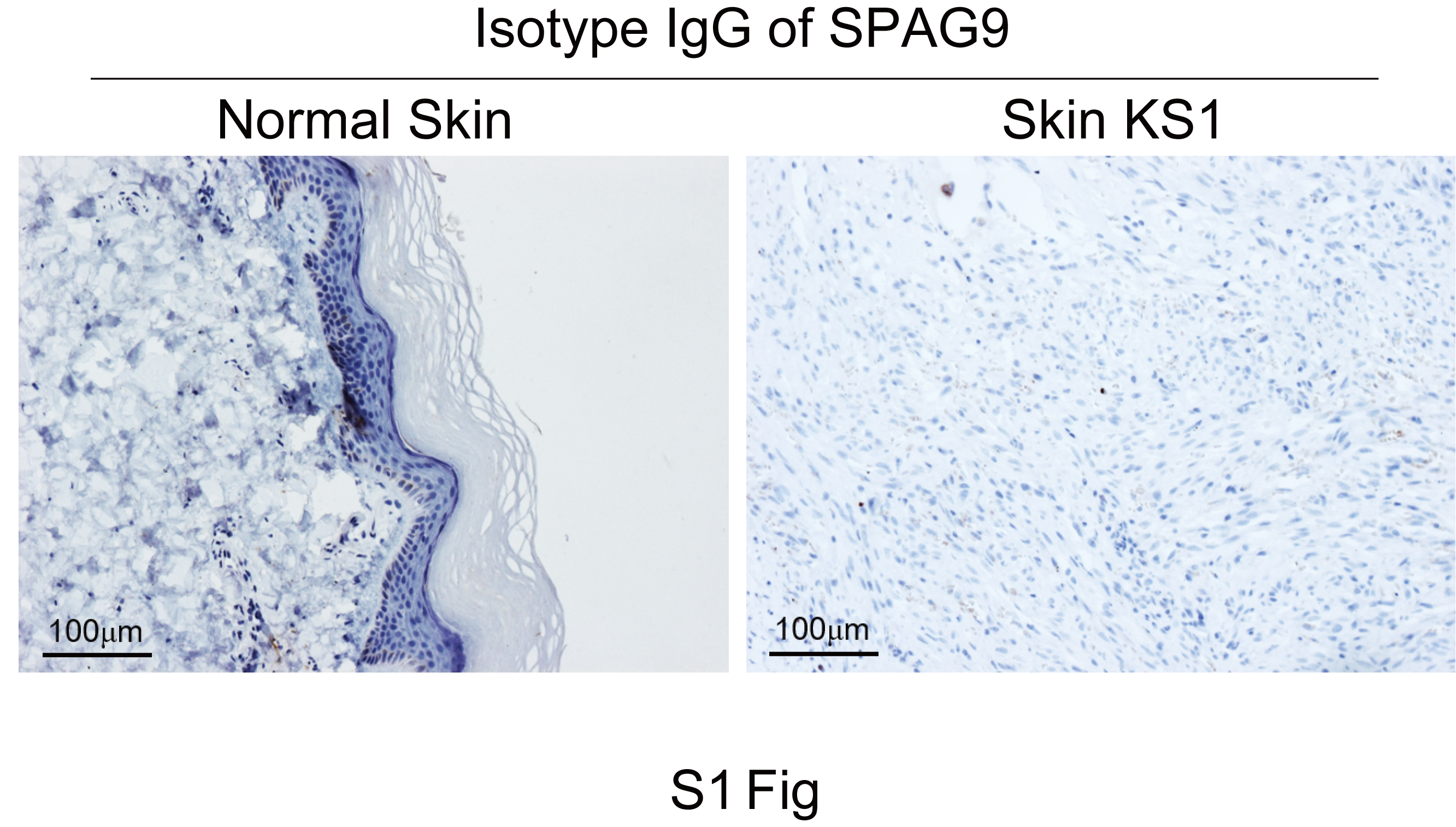

Supplement: S1 Fig — Immunohistochemical staining of isotype control immunoglobulin G (IgG) in normal skin, and skin KS of patient #1 (Skin KS1). Magnification, ×200. (TIF) [file ppat.1008730.s001.tif]

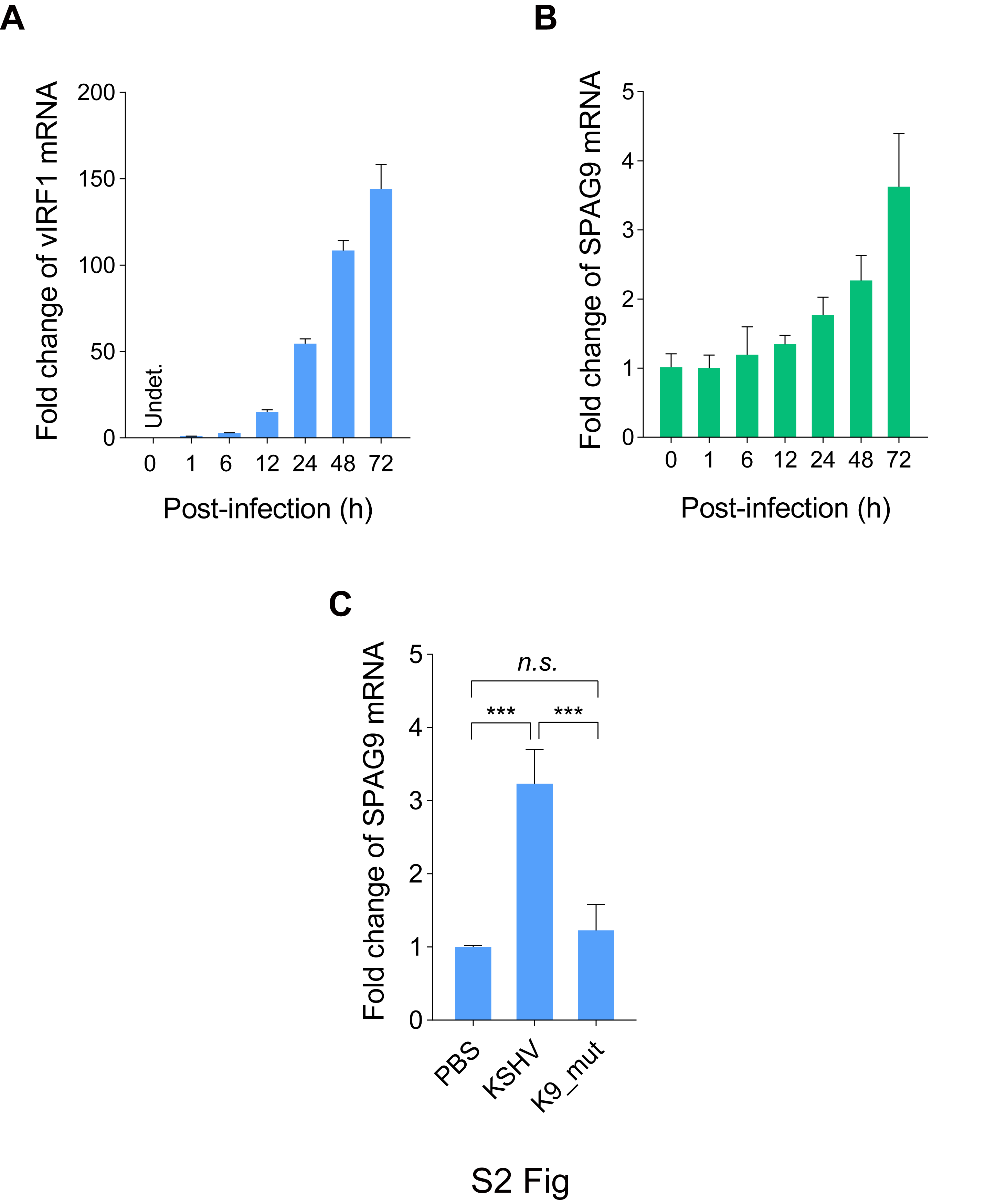

Supplement: S2 Fig — (A). RT-qPCR analysis of mRNA level of vIRF1 in HUVECs infected by KSHV wild-type virus (KSHV) (MOI of 3). Samples were collected at 0 h, 1 h, 6 h, 12 h, 24 h, 48 h, and 72 h after KSHV infection. Undet., undetermined. (B). RT-qPCR analysis of mRNA level of SPAG9 in HUVECs infected by KSHV wild-type virus (KSHV) (MOI of 3). Samples were collected at 0 h, 1 h, 6 h, 12 h, 24 h, 48 h, and 72 h after KSHV infection. (C). Lack of vIRF1 reduces SPGA9 mRNA transcript induced by KSHV. RT-qPCR analysis of mRNA level of SPAG9 in HUVECs treated with PBS (PBS) or infected with wild-type KSHV (KSHV_WT) (MOI of 3) or vIRF1 mutant virus (K9_mut) (MOI of 3) for 30 h. Data were shown as mean ± SD. *** P < 0.001, Student's t-test. n.s, not significant. (TIF) [file ppat.1008730.s002.tif]

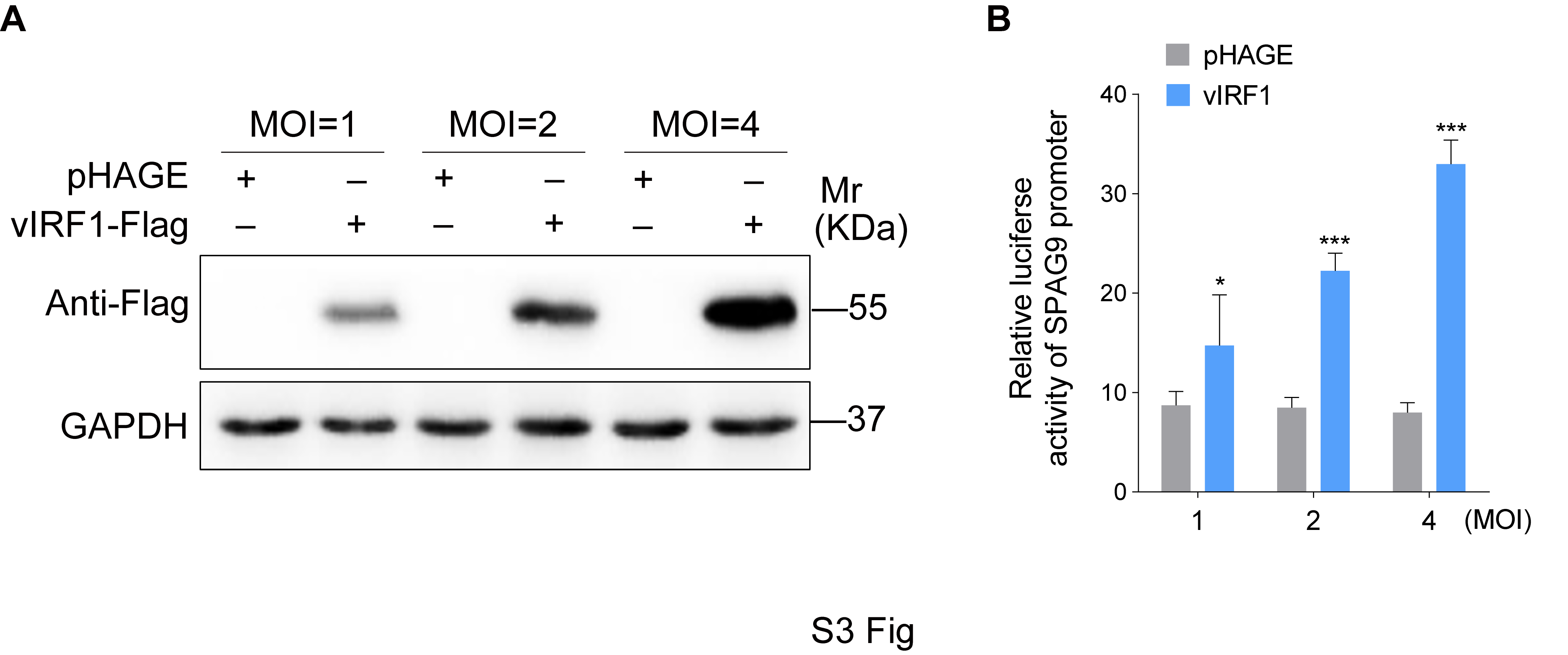

Supplement: S3 Fig — (A). Western-blotting analysis of vIRF1 (Anti-Flag) in HUVECs transduced with different MOIs (1, 2 and 4) of lentiviral-vIRF1 or its control lentiviral-pHAGE. (B). Luciferase reporter assay of the activity of SPAG9 promoter in HUVECs transduced with different MOIs (1, 2 and 4) of lentiviral-vIRF1 or its control lentiviral-pHAGE. Data were shown as mean ± SD. * P < 0.05 and *** P < 0.001, Student's t-test. (TIF) [file ppat.1008730.s003.tif]

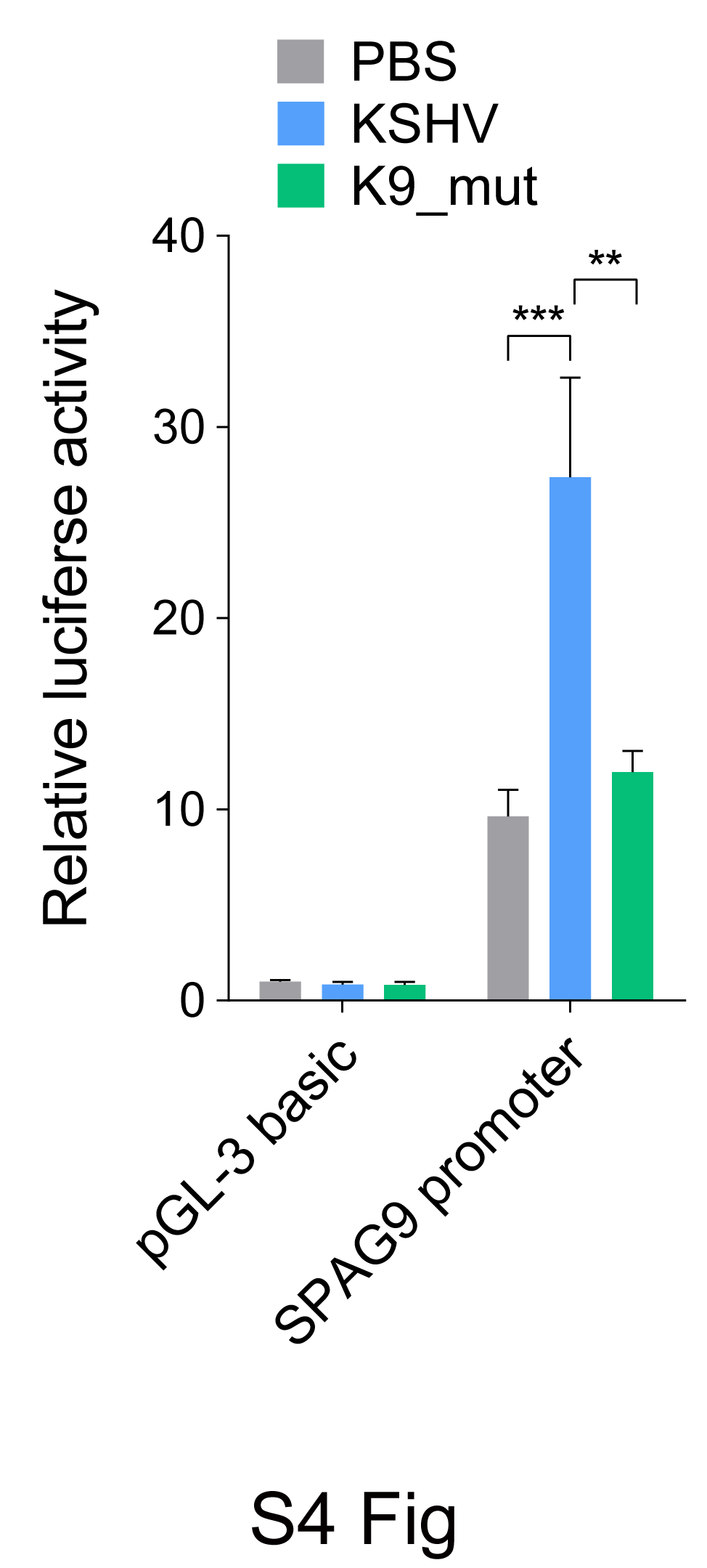

Supplement: S4 Fig — Luciferase reporter assay of the activity of SPAG9 promoter in HUVECs treated with PBS (PBS) or infected with wild-type KSHV (KSHV_WT) (MOI of 3) or vIRF1 mutant virus (K9_mut) (MOI of 3) for 30 h. Data were shown as mean ± SD. ** P < 0.01 and *** P < 0.001, Student's t-test. (TIF) [file ppat.1008730.s004.tif]

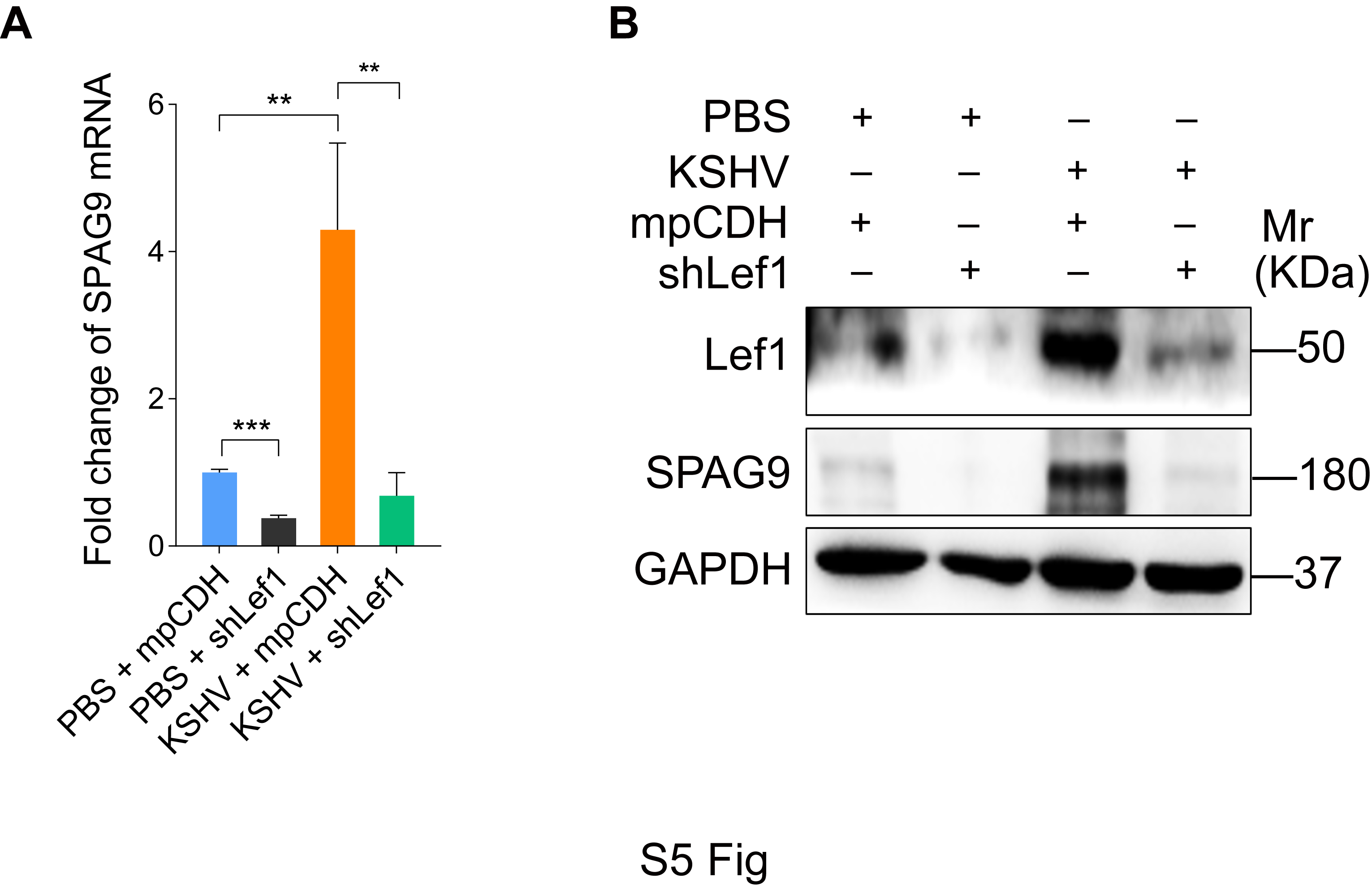

Supplement: S5 Fig — (A). RT-qPCR analysis of mRNA level of SPAG9 expression in KSHV-infected HUVECs transduced with a mixture of lentivirus-mediated shRNAs targeting Lef1 (shLef1). Data were shown as mean ± SD. ** P < 0.01 and *** P < 0.001, Student's t-test. (B). Western-blotting analysis of SPAG9 expression in KSHV-infected HUVECs transduced with a mixture of lentivirus-mediated shRNAs targeting Lef1 (shLef1). (TIF) [file ppat.1008730.s005.tif]

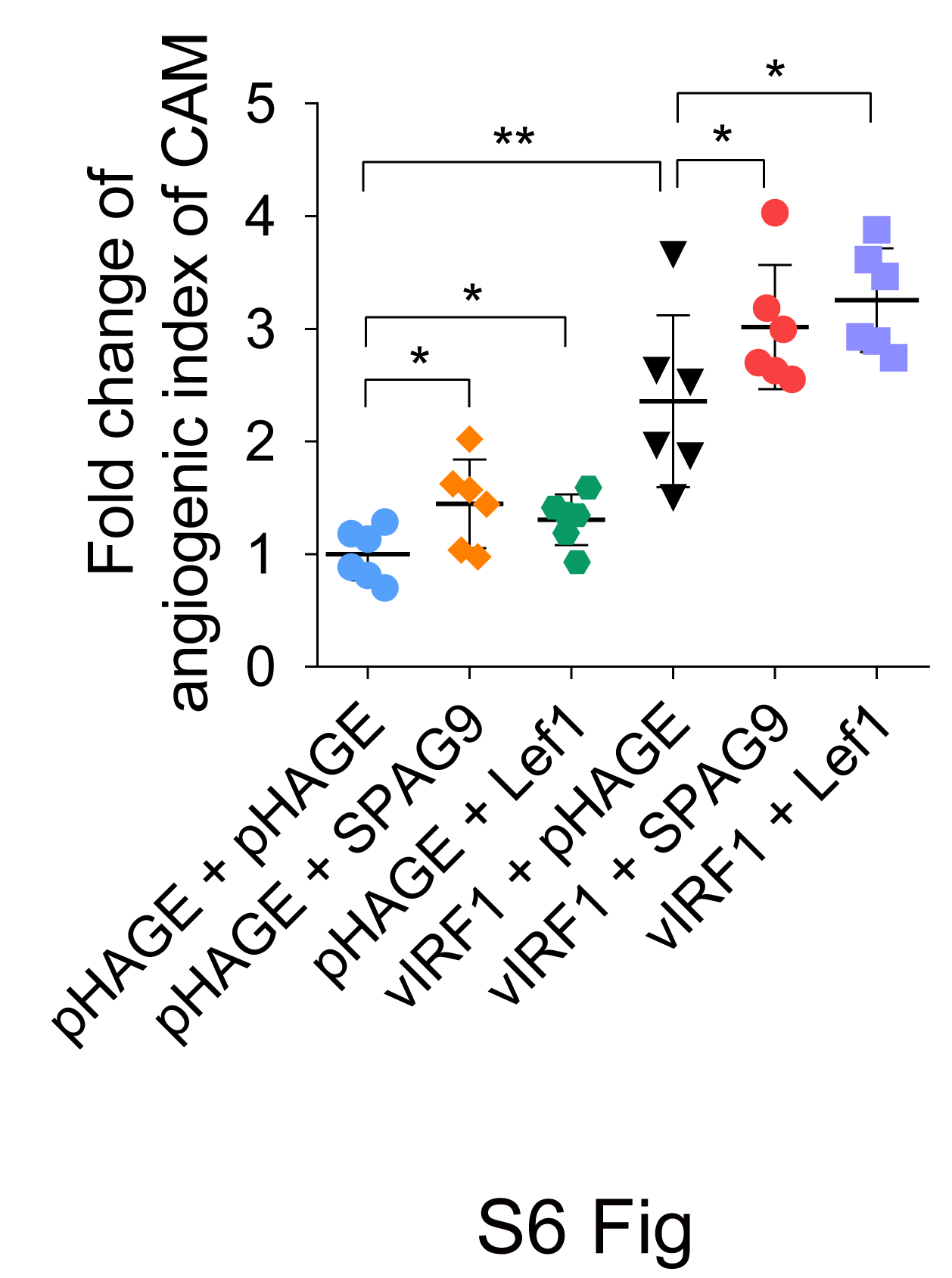

Supplement: S6 Fig — Lentiviral vIRF1- or its control pHAGE-infected endothelial cell line were transduced with lentivirus-SPAG9 (SPAG9), lentivirus-Lef1 (Lef1) or its control pHAGE (pHAGE), respectively, and then were subjected to chicken chorioallantoic membranes (CAMs) assay. Quantification of CAMs assay was showed. Data were shown as mean ± SD. * P < 0.05 and ** P < 0.01, Student's t-test. (TIF) [file ppat.1008730.s006.tif]

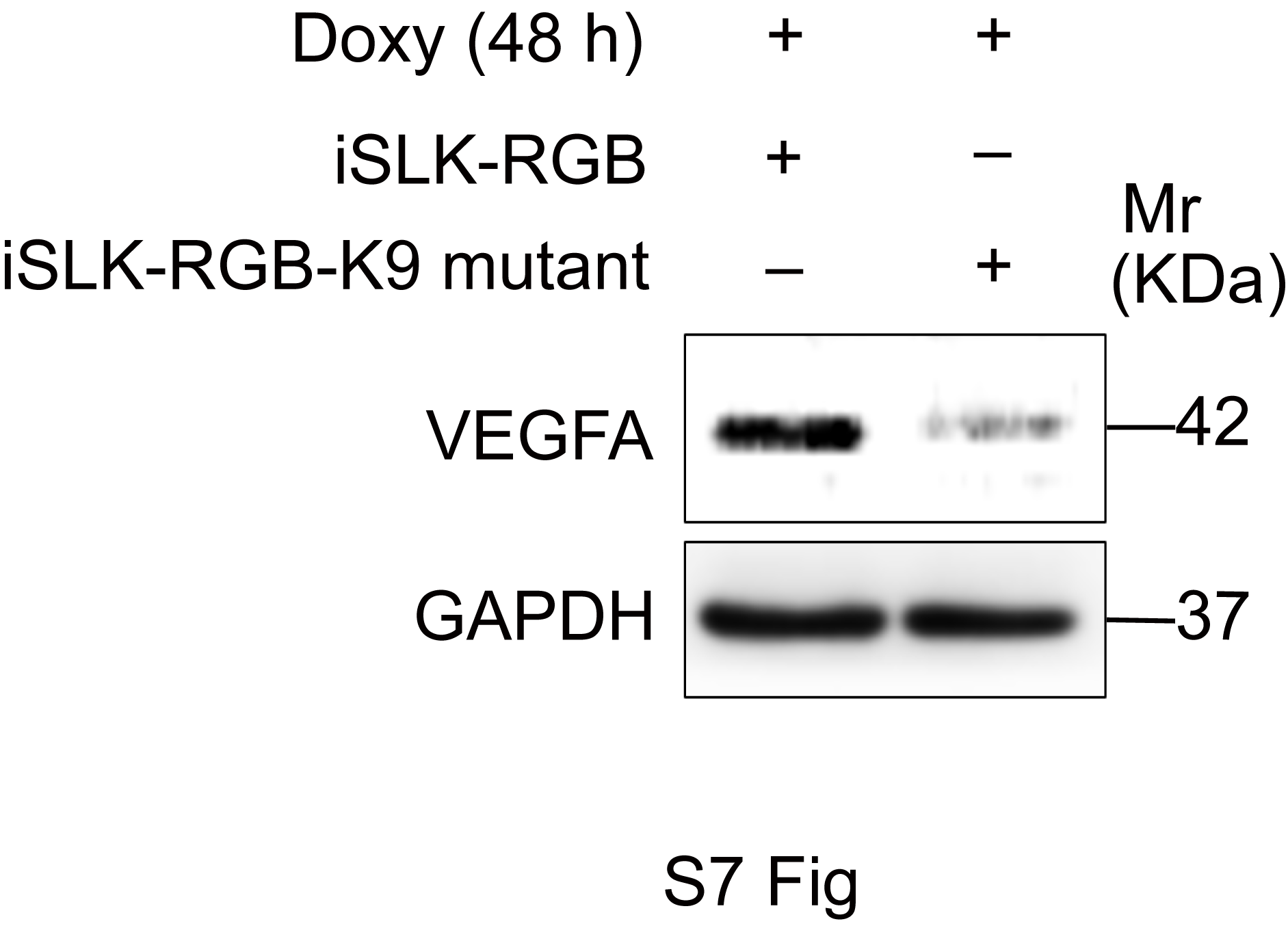

Supplement: S7 Fig — Western-blotting analysis of VEGFA expression in iSLK-RGB cells and iSLK-RGB-K9 mutant cells treated with doxycycline (Doxy) (1 μg/ml) for 48 h. (TIF) [file ppat.1008730.s007.tif]

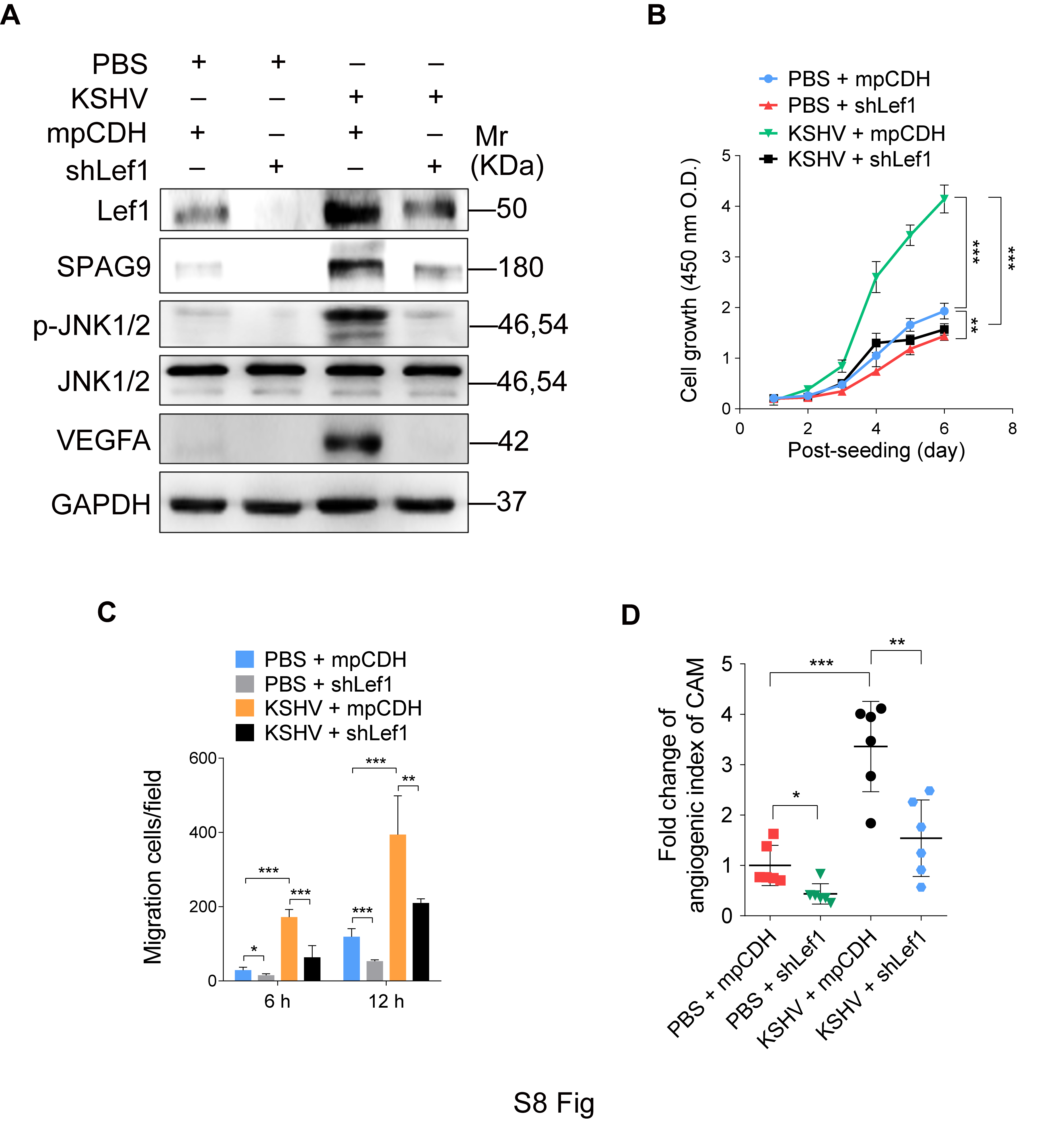

Supplement: S8 Fig — (A). Western-blotting analysis of the expressions of SPAG9, p-JNK1/2, and VEGFA in KSHV-infected HUVECs transduced with a mixture of lentivirus-mediated shRNAs targeting Lef1 (shLef1). (B). CCK-8 assay of HUVECs treated as in (A). (C). Transwell migration analysis of HUVECs treated as in (A). The migrated HUVECs were counted at 6 h and 12 h post seeding. (D). PBS-treated or KSHV-infected endothelial cell line were transduced with a mixture of lentivirus-mediated shRNAs targeting Lef1 (shLef1) for 48 h, and then were subjected to chicken chorioallantoic membranes (CAMs) assay. Data were shown as mean ± SD. * P < 0.05, ** P < 0.01 and *** P < 0.001, Student's t-test. (TIF) [file ppat.1008730.s008.tif]

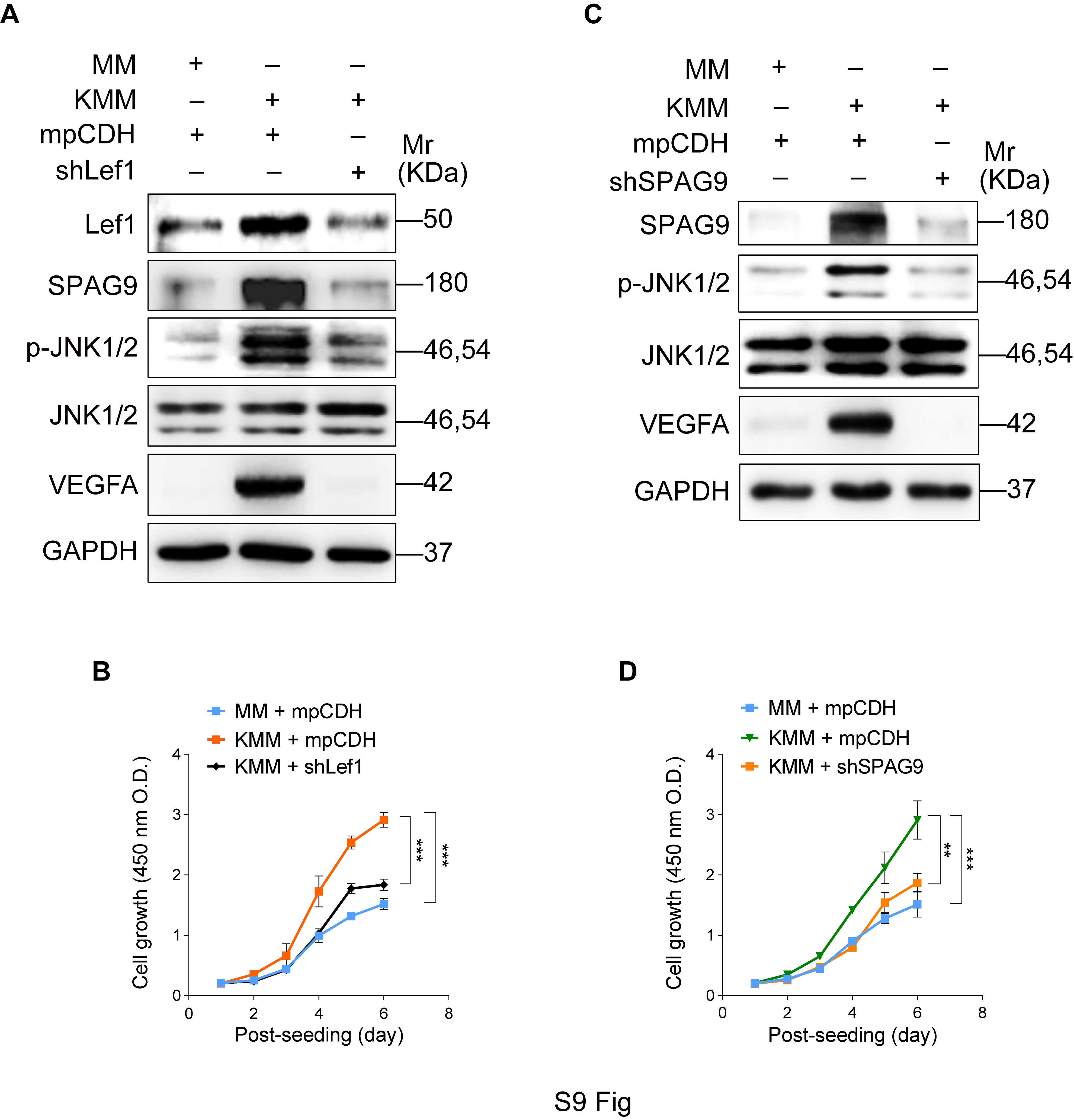

Supplement: S9 Fig — (A). Western-blotting analysis of Lef1, SPAG9, p-JNK1/2 and VEGFA expression in MM and KMM cells transduced with a mixture of lentivirus-mediated shRNAs targeting Lef1 (shLef1). (B). CCK-8 assay of cells treated as in (A). (C). Western-blotting analysis of SPAG9, p-JNK1/2 and VEGFA expression in MM and KMM cells transduced with a mixture of lentivirus-mediated shRNAs targeting SPAG9 (shSPAG9). (D). CCK-8 assay of cells treated as in (C). Data were shown as mean ± SD. ** P < 0.01 and *** P < 0.001, Student's t-test. (TIF) [file ppat.1008730.s009.tif]
